# *In Vivo* and *In Vitro* Pharmacokinetic Studies of a Dual Topoisomerase I/II Inhibitor

**DOI:** 10.1021/acsptsci.4c00596

**Published:** 2025-03-12

**Authors:** Jonas Hildebrandt, Dirk O. Bauerschlag, Gert Fricker, Ulrich Girreser, Björn Konukiewitz, Franziska Kellers, Nicolai Maass, Bernd Clement, Inken Flörkemeier

**Affiliations:** † 9179 Christian-Albrechts-University Kiel, Pharmaceutical Institute, Department of Pharmaceutical and Medicinal Chemistry, Kiel 24118, Germany; ‡ Department of Gynaecology and Obstetrics, 54186 University and University Medical Center Schleswig-Holstein Campus Kiel, Kiel 24105, Germany; § 9144 Ruprecht-Karls University, Institute of Pharmacy and Molecular Biotechnology, Heidelberg 69120, Germany; ∥ Department of Pathology, 15056 University and University Medical Center Schleswig-Holstein Campus Kiel, Kiel 24105, Germany; ⊥ Department of Gynecology and Reproductive Medicine, Jena University Hospital, Jena 07747, Germany

**Keywords:** metabolism, bioavailability, cancer, dual topoisomerase inhibitor, P8-D6, pharmacokinetic studies, protein binding, distribution, elimination, P-GP, BCR

## Abstract

Due to high mortality rates, new and more effective drugs are urgently needed in cancer therapy. The novel dual topoisomerase inhibitor P8-D6, a dimethylaminoethyl-substituted pyridophenanthroline, showed *in vitro* impressive induction of apoptosis in tumors such as ovarian cancer or multiple myeloma compared to the current standard therapy. The purpose of this study was to investigate its *in vitro* and *in vivo* pharmacokinetics and to discover further potential drug candidates. Samples of plasma, various tissues, urine, feces, and cell culture supernatants were examined by HPLC. In addition, the efficacy of the metabolites against ovarian cancer was determined *in vitro*. Three phase I metabolites were identified *in vitro* and *in vivo*, and one phase II metabolite was identified *in vivo*. Among the metabolites, *N*-dealkylated P8-D6 (P8-D6 mono) achieved efficacy similar to that of P8-D6 in ovarian cancer. P8-D6 showed a relevant inhibitory effect on the efflux pumps P-GP (IC_50_ = 20.63 μM) and BCRP (16.32 μM). The calculated oral bioavailability in Sprague–Dawley rats was 21.5%, while P8-D6 had a high plasma protein binding of 99% and an extensive tissue distribution with an apparent volume of distribution between 57.69 (i.v.) and 82.92 (p.o.) L/m^2^. Both P8-D6 and its metabolites were detected in urine and feces. This study provides a basis for the clinical application of P8-D6 and has also identified P8-D6 mono as a very potent and metabolically stable drug candidate.

## Graphical abstract

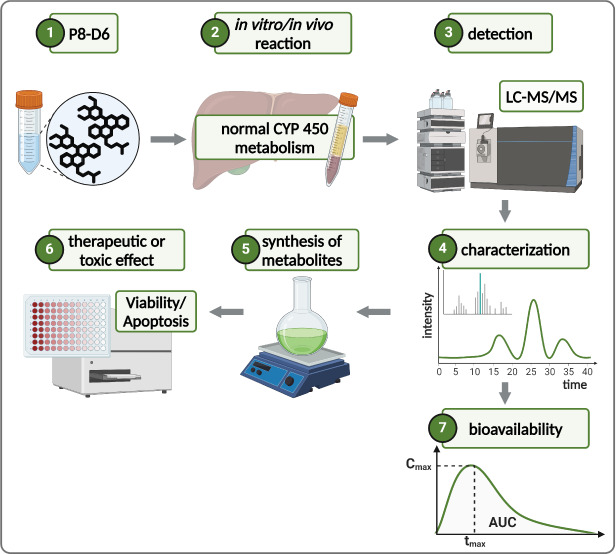

Because ovarian cancer is frequently diagnosed at an advanced stage, it is the fifth-leading cause of death among female cancer patients in the USA and EU.^1,2^ The standard therapy regimen currently consists of cytoreductive surgery followed by platinum-based chemotherapy, with the option of maintenance therapy with bevacizumab and/or PARP inhibitors.^3,4^ Nevertheless, many patients suffer a recurrence despite advances in therapy.^5,6^ Second-line therapies include the topoisomerase I inhibitor topotecan or pegylated liposomal doxorubicin.^7,8^ However, these treatments unfortunately do not lead to significantly prolonged progression-free survival.^9,10^ Consequently, the search for new effective therapy options is of high scientific and medicinal interest.

Topoisomerases I and II are essential for DNA replication, transcription, and cell proliferation by maintaining DNA topology.^11–13^ Inhibition of topoisomerase I leads to DNA single-single strand breaks, while inhibition of topoisomerase II induces double-strand breaks, both resulting in apoptosis.^11,14–16^ In response to drug-induced inhibition of one of the topoisomerase forms, an upregulation of the unaffected topoisomerase leads to some extent of resistance. Dual topoisomerase I/II inhibitors represent a novel therapy option to counter this mechanism.^10,11,14,17–19^ Although this approach offers appealing pharmacological options, and therefore, several drug candidates like TAS-103, intoplicine, pyrazoloacridine, elomotecan, F-11782, XR11576, and XR-5000 have been developed, none of them have yet been approved due to severe side effects.^17,20–29^

A promising new agent for the treatment of ovarian cancer is the aza-analogous benzo­[*c*]­phenanthridine P8-D6 (6-(*N,N*-dimethyl-2-aminoethoxy)-11-(3,4,5-trimethoxyphenyl)­pyrido­[3,4-*c*]­[1,9]­phenanthroline), which was synthesized in an optimized four-step process with advantageous physicochemical and cytotoxic properties.^30^ P8-D6 is a dual topoisomerase (I/II) inhibitor as it stabilizes the covalent DNA-topoisomerase intermediate of both topoisomerases.^31^ Its high antitumoral efficacy in the therapy of ovarian cancer, breast cancer, and multiple myeloma was recently demonstrated in *in vitro* and *in vivo* studies.^5,32,33^ Studies on combination therapy with the established PARP inhibitor olaparib led to an accumulation of DNA damage and a sensitization of cancer cells to olaparib.^34^ Based on these promising results and to push forward its drug development, the pharmacokinetics of P8-D6 was intensively investigated in this study.

Pharmacokinetic studies are mandatory in preclinical and clinical drug development. In this work, both *in vitro* and *in vivo* studies were conducted to extensively describe the pharmacokinetics of P8-D6. A suitable HPLC and LC-MS method for the quantification of P8-D6 and its metabolites in biological samples was developed and validated. Initially, the *in vitro* metabolism of P8-D6 was investigated using tissue material and subcellular fractions from pigs, mice, rats, and humans and human ovarian cancer cell lines. A targeted examination of the enzymatic conversion was carried out using recombinant CYPs and FMO3. Cytochrome P450 (CYP) enzymes, consisting of a superfamily of oxidoreductases, and flavin-containing monooxygenases (FMO) possess relevance for the phase I metabolism of xenobiotics.^35,36^

Furthermore, this work focused on the *in vivo* bioavailability and metabolism of P8-D6 after oral (p.o.) and intravenous (i.v.) administration in rats. This included the distribution based on pharmacokinetic parameters and *in vivo* biotransformation in phases I and II in plasma, tissue materials, and excretions. Since plasma protein binding, interaction with transporter proteins, and metabolizing enzymes can also have an influence on efficacy, these pharmacokinetic parameters were also determined. Finally, all discovered metabolites were chemically synthesized and characterized with regard to efficacy. One highlight of this study is the identification of a highly effective metabolite that could become a promising drug candidate.

## Results

In this work, analytical methods using HPLC, LC-MS, and NMR were developed to identify and quantify P8-D6 and its metabolites *in vitro* and *in vivo*. The HPLC method was validated in terms of range (0.5–25 μM, 1.29–88 nmol/g), linearity (*R* ^2^ > 0.999), average accuracy (99.37%), precision (±2.29%), and recovery (97.7%) (supplied as Supporting Information Chapter 2).

### *In Vitro* Biotransformation Studies

P8-D6 (1 mM) was incubated with postnuclear supernatant (PNS), microsomes, or S9 reaction mixture of various tissue samples (liver, kidney, lung, spleen, and colon) from different donor species (pigs, mice, rats, and humans) for 150 min (study preparation scheme supplied as Figure S5). The commercial human and rat enzymes were subjected to quality control by the supplier. Three phase I metabolites were identified. The HPLC chromatogram and the structures of P8-D6 and its detected metabolites are shown in Figure 1. This shows that all reactions involve the side chain. LC-MS and NMR spectroscopic data for the metabolites are given in Supporting Information. Based on the structural characteristics, the metabolites were named as follows: P8-DO (**4**), P8-D6 *N-*oxide (**6**), and P8-D6 mono (**8**). All metabolites were synthesized to confirm the postulated structure (chapter Synthesis of Metabolites). The extent of metabolism during *in vitro* incubation was highly dependent on the species (pig > mouse > rat > human) (Figure 2).

**Figure 1:**
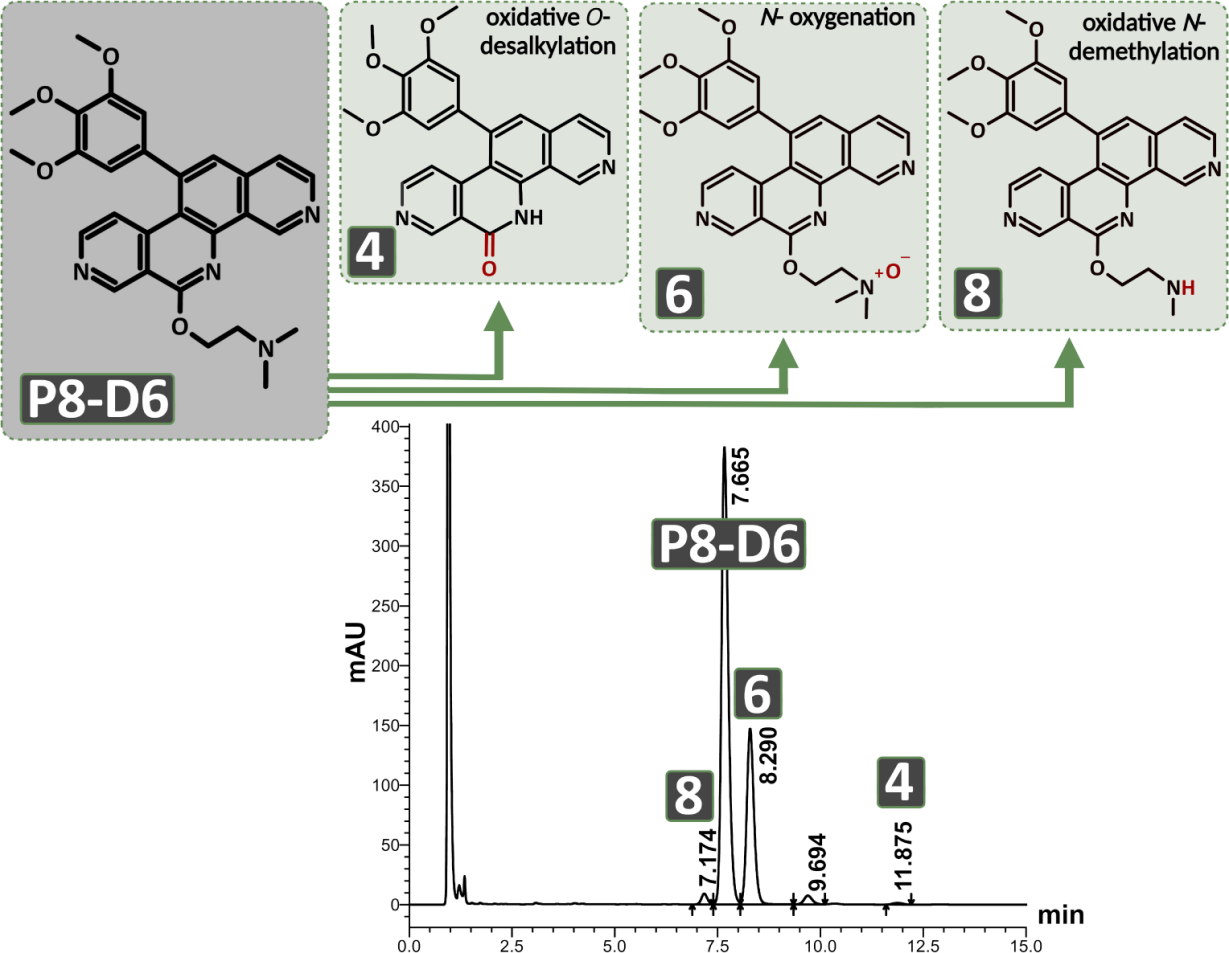
P8-D6 and its detected metabolites and a representative HPLC chromatogram after incubation, showing all derivatives. P8-DO (**4**), P8-D6 *N*-oxide (**6**), and P8-D6 mono (**8**). Substances were analyzed under the following conditions: C18 column, flow rate 0.9 mL/min, isocratic solvent 28% acetonitrile, 72% 100 mM ammonium acetate (pH 4.5), detection 260 nm.

**Figure 2:**
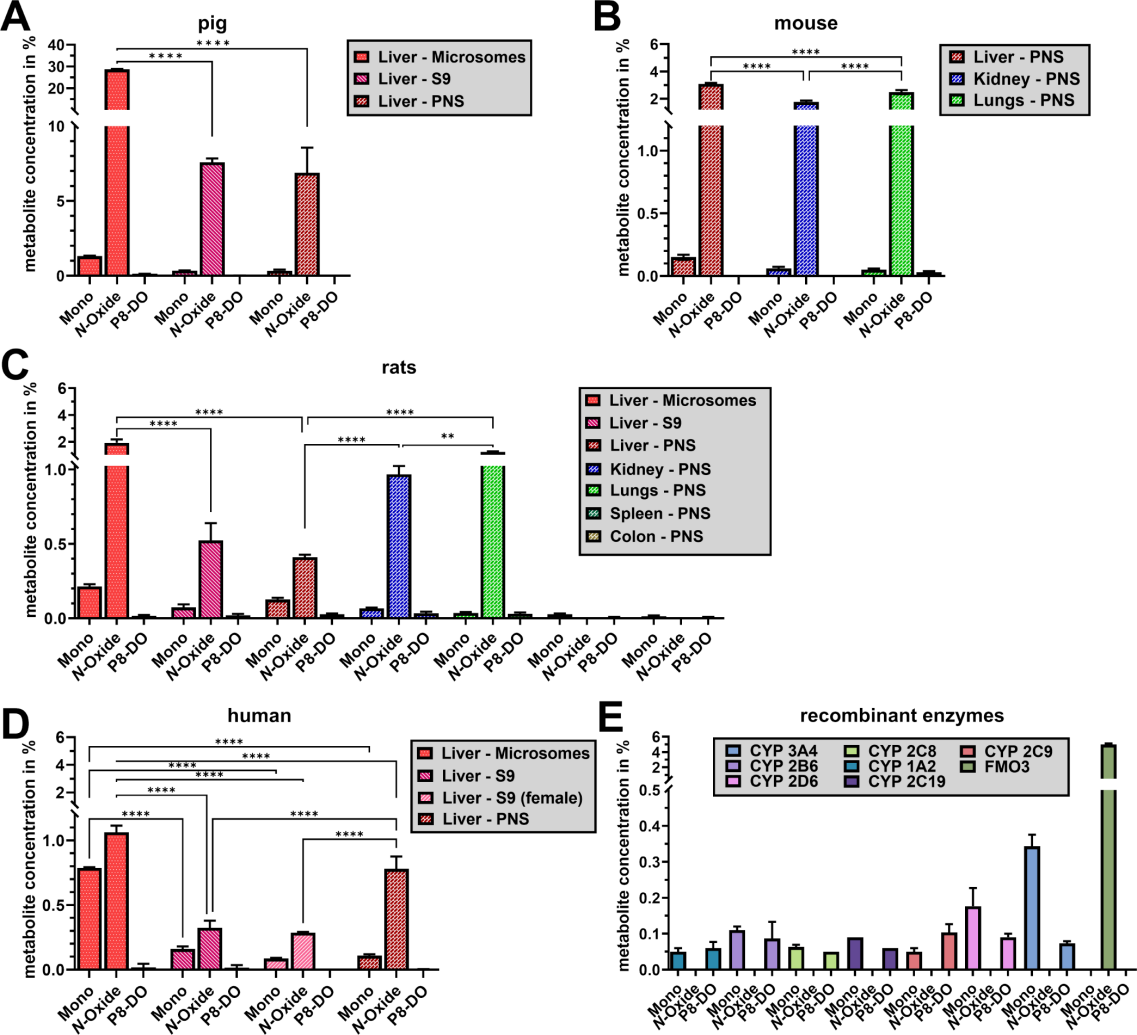
*In vitro* biotransformation of P8-D6 in homogenized tissue of the liver, kidney, lung, spleen, and/or colon. The tissues of the different animal species pig (A), mouse (B), rat (C), and human (D) were fractionated into the PNS, S9 fraction, and microsomal fraction and incubated with P8-D6. The detection of the responsible enzymes was performed by enzymatic conversion of P8-D6 with recombinant metabolic enzymes (E). The quantification was carried out using HPLC. Data are means + SD (*n* ≥ 3). Data are normally distributed (Shapiro–Wilk test), one-way ANOVA (Tukey’s multiple comparisons test); *p < 0.05, **p < 0.01, ***p < 0.001, ****p < 0.0001. One-way ANOVA, *p < 0.05, **p < 0.01, ***p < 0.001, ****p < 0.0001.

P8-D6 *N*-oxide was the most common metabolite for all incubations with subcellular fractions from the homogenized tissue. According to the detected MS-MS fragments (supplied as Supporting Information Chapter 2), it resulted from *N*-oxygenation of the nitrogen at the side chain since the unmodified fragment of P8-DO (*m*/*z* = 414.1) was detected. Furthermore, the detected fragment at *m*/*z* = 440.1 is most likely a carbocation at the remaining ethyl chain after splitting off the dimethylamine *N*-oxide further strengthening the theory of selectively forming this specific *N*-oxide. In addition, no relevant chemical shifts in the aromatic range of the ^1^H NMR compared to P8-D6 and mono were observed (supplied as Supporting Information Chapter 2). Thus, no chemical modifications can have occurred on the aromatic rings, and all changes must therefore affect the side chain. The overall greatest extent of metabolism was observed in the pig liver (pig > mouse > rat > human). Among their subcellular fractions, liver microsomes yielded the most formation at 28.67% P8-D6 *N*-oxide, significantly exceeding the S9-fraction (7.58%) and PNS (6.88%) (Figure 2 A). Comparing the conversion rates of liver microsome fractions from different species, substantially less *N*-oxide was detected in rat (1.91%) and human tissue (1.07%) (Figure 2 C,D). In liver PNS of mice with 3.08%, the second most formation of P8-D6 *N*-oxide was observed, followed by humans and rats (Figures 2 B–D). The human S9 liver fraction from mixed-sex and female donors showed no significant differences (*p* > 0.74). Analysis of the PNS fraction in various metabolic organs of the rat determined the highest metabolic rate in the lungs (1.37%), followed by the kidney and the liver, and a low proportion in the spleen and colon (Figure 2C). To determine which enzymes were responsible for the conversion, seven recombinant CYP 450 isoenzymes recommended by the FDA’s “Guidance for Industry” and human FMO3 were used. When incubated with recombinant enzymes, P8-D6 was metabolized to P8-D6 *N*-oxide only with FMO3 (Figure 2E).

The second most common *in vitro* metabolite of P8-D6 was P8-D6 mono. It resulted from an oxidative *N*-demethylation of one methyl group at the side chain (Figure 1). The extent of formation of P8-D6 mono was 21.9-fold lower than P8-D6 *N*-oxide, reaching a maximum of only 1.31% in porcine liver microsomes (Figure 2A). Similar to P8-D6 *N*-oxide, in all species, the formation of P8-D6 mono was highest with liver microsomes (Figure 2). However, among all species and organs, P8-D6 mono relatively occurred the most in the human liver. When comparing the organs, the liver was the strongest metabolic organ (Figure 2 B,C).

Among the recombinant enzymes, P8-D6 is mostly metabolized to P8-D6 mono by CYP3A4 (0.34%, significant compared to any other enzyme, *p* < 0.0001) and 2D6 (0.17%, significant compared to 2C8, 1A2, 2C19, and 2C9, *p* < 0.005) (Figure 2 E).

The third and final *in vitro* metabolite, P8-DO, is a direct synthesis precursor of P8-D6 and results from oxidative O-desalkylation of the complete side chain (Figure 1). However, this metabolite does not play a prominent role in *in vitro* biotransformation, as it only results in a maximum formation of 0.13% with pig liver microsomes (Figure 2 A). The formation of P8-DO is mainly catalyzed by CYP2C9 (0.10%) and 2D6 (0.09%) (Figure 2 E).

### *In Vitro* CYP Inhibition

To assess metabolizing enzyme-mediated drug interactions, P8-D6 was incubated with human liver (HLM) or recombinant CYP enzymes and the respective marker substrate according to the FDA (with the exception of using amitriptyline instead of *S*-mephenytoin).^37–39^

The highest impact on the CYP activity was observed for CYP 3A4 and 2C9 for both HLM and recombinant CYP, as well as 2C19 (HLM) and 2D6 (recombinant enzyme; Table 1, supplied as Figure S1). Since the HLM had a lower CYP concentration than the respective recombinant enzyme, conversion rates were lower. However, as some conversions were catalyzed by several enzymes, the conversion rates in HLM increased (e.g., bupropion and diclofenac).

**Table 1:** IC_50_ Values of the CYP Inhibition by P8-D6

	(IC_50_ in μM)	
CYP	HLM	CYP isoenzyme
Phenacetin (CYP1A2)	912.5	503.1
Bupropion (CYP2B6)	^a^	^a^
Amodiaquine (CYP2C8)	245.5	282.1
Diclofenac (CYP2C9)	162.1	71.44
Amitriptyline (CYP2C19)	148.7	792.8
Dextromethorphan (CYP2D6)	285.0	166.1
Testosterone (CYP3A4)	164.8	94.03

a = no inhibition detected.

### Synthesis

P8-DO (**4**), P8-D6 *N*-oxide (**6**), and P8-D6 mono (**8**) were synthesized according to Scheme 1.

**Scheme 1:**
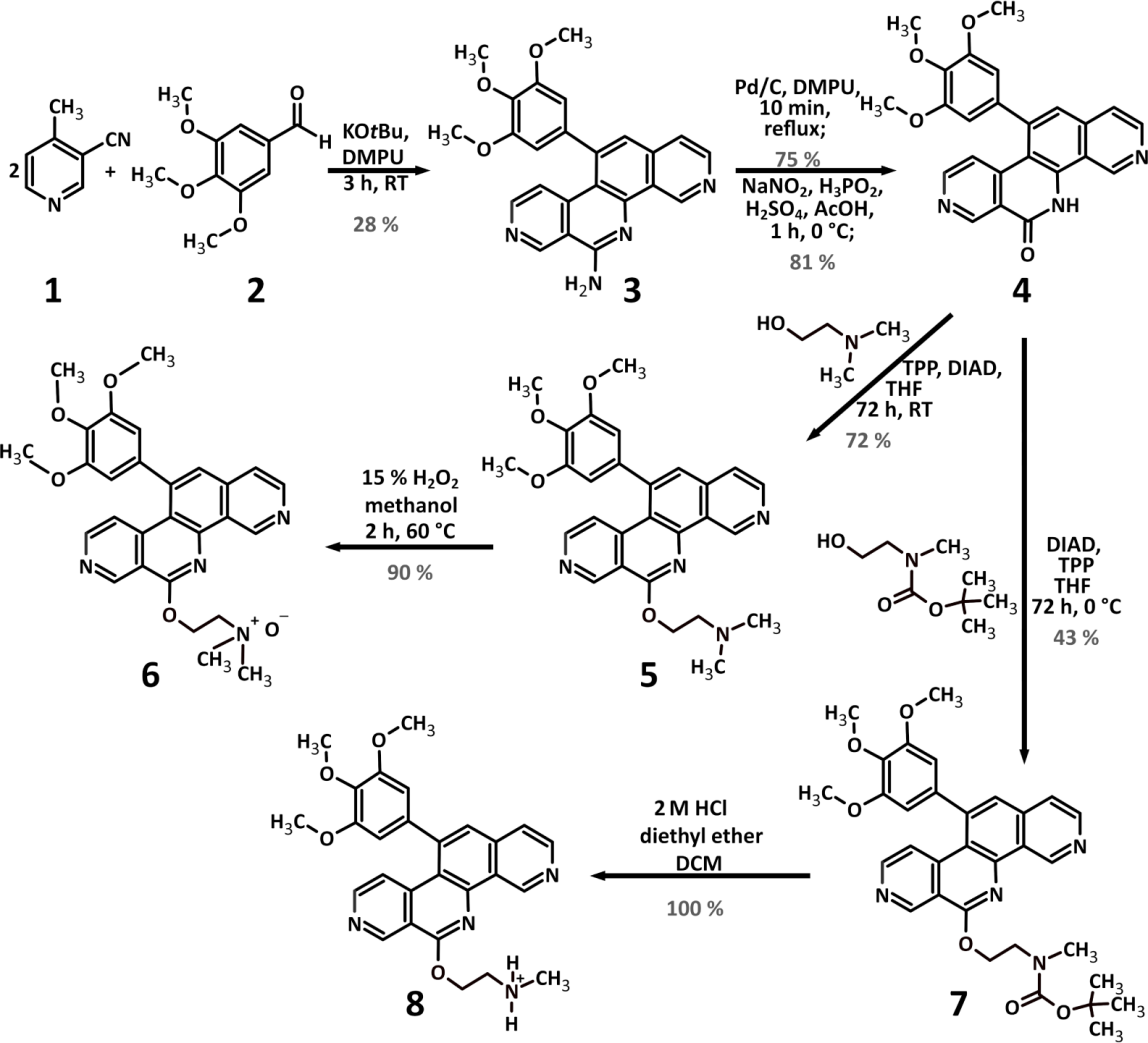
Synthesis of P8-D6 (**5**) and Its Metabolites P8-DO (**4**), P8-D6 *N*-Oxide (**6**), and P8-D6 Mono (**8**)­^a^

a Synthesis of P8-DO (**4**), P8-D6 *N*-oxide (**6**), and P8-D6 mono (**8**). KO*t*Bu (potassium *tert*-butoxide), Pd/C (palladium on carbon), NaNO_2_ (sodium nitrite), H_3_PO_2_ (hypophosphorous acid), H_2_SO_4_ (sulfuric acid), AcOH (acetic acid), TPP (triphenylphosphine), DIAD (diisopropyl azodicarboxylate), H_2_O_2_ (hydrogen peroxide), and HCl (hydrochloric acid).

P8-D6 (**5**) was synthesized using a simple and optimized four-step approach according to Meier et al.^30^ Since P8-DO (**4**) is a synthesis precursor of P8-D6 (**5**), it was easily obtained during synthesis. Likewise, the synthesis of P8-D6 mono (**8**) started from P8-DO (**4**). The final Mitsunobu reaction required the use of *N*-Boc protected *N*-methylaminoethanol, as otherwise a reactive secondary amine would be formed. The protective group was removed with 2 M HCl in diethyl ether, leading to the precipitation of the final protonated product.

P8-D6’s structure generally offers multiple nitrogen atoms for *N*-oxygenation. However, according to MS-MS fragments of the metabolite P8-D6 *N*-oxide (**6**), the *N-*oxygenated nitrogen atom was identified as the side chain nitrogen. Starting from a solution of the free base of P8-D6 in methanol, the synthesis of P8-D6 *N*-oxide (**6**) was achieved by adding 15% aqueous H_2_O_2_ at 65 °C.

### Cell Culture

The efficacy of metabolites can have a significant impact on the efficacy of the drug. Due to the low *in vitro* formation of P8-DO and based on the NCI 60 Cell Screening, it proved to be ineffective against cancer (supplied as Figure S2) and, therefore, was not tested in the cell culture. The therapeutic effect of P8-D6 mono and P8-D6 *N*-oxide was investigated *in vitro* in human ovarian cancer 2D monolayers and 3D spheroids (Figures 3 and 4). 48 h of treatment of the ovarian cancer cell lines (OvCar8, A2780, Igrov-1) with P8-D6 mono and P8-D6 *N*-oxide was compared to P8-D6 and cisplatin (study preparation scheme supplied as Figure S5). Both metabolites decreased viability in ovarian cancer cells in a 2D monolayer (Figure 3 A, supplied as Figure S3 A,C). However, P8-D6 mono (IC_50_ = 0.31 μM) was just as effective as P8-D6 (IC_50_ = 0.27 μM). P8-D6 *N*-oxide obtained an IC_50_ value of 1.98 μM. The apoptosis induction of P8-D6 mono was comparable to that of P8-D6 (Figure 3 B, supplied as Figure S3 B,D). P8-D6 *N*-oxide, on the other hand, only differed significantly (3.5-fold higher apoptosis rate) from cisplatin at a concentration of 10 μM.

**Figure 3:**
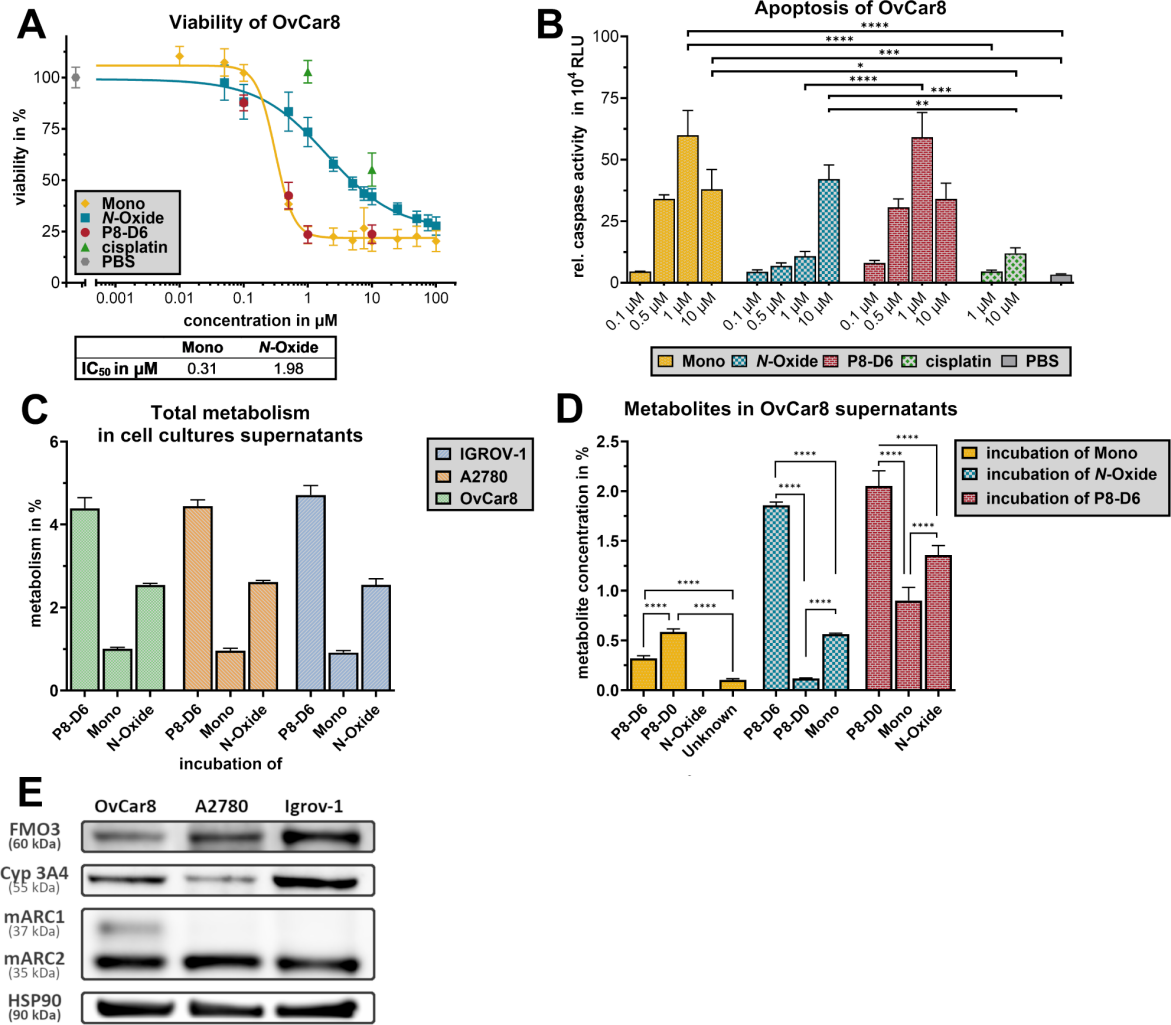
Antitumoral effect of P8-D6 mono and P8-D6 *N*-oxide in ovarian cancer 2D monolayers. OvCar8 cells were treated for 48 h with of P8-D6 mono, P8-D6 *N-*oxide, P8-D6, cisplatin, and PBS. Subsequently, the viability (A) and caspase activity (B) were measured. The IC_50_ value of P8-D6 mono and P8-D6 *N-*oxide was calculated using the viability data (A). The enzymatic conversion of P8-D6 into its metabolites was analyzed in different human ovarian cancer cell lines by examining the supernatant of this cell culture for the metabolites formed. The total metabolism (as the sum of all detected metabolites as a percentage of the substrate) after incubation with P8-D6, P8-D6 mono, and P8-D6 *N*-oxide (without identification of the metabolites) is shown for OvCar8, A2780, and Igrov-1 (C). For OvCar8 supernatants, the resulting metabolites were identified and quantified after incubation with P8-D6, P8-D6 mono, and P8-D6 *N*-oxide (D). Data are means ± SEM (*n* = 3). Data are normally distributed (D’Agostino and Pearson test), ordinary one-way ANOVA (Šídák’s multiple comparisons test); *p < 0.05, **p < 0.01, ***p < 0.001, ****p < 0.0001. (E) Western blot for detection of enzyme expression in the untreated cell lines.

**Figure 4:**
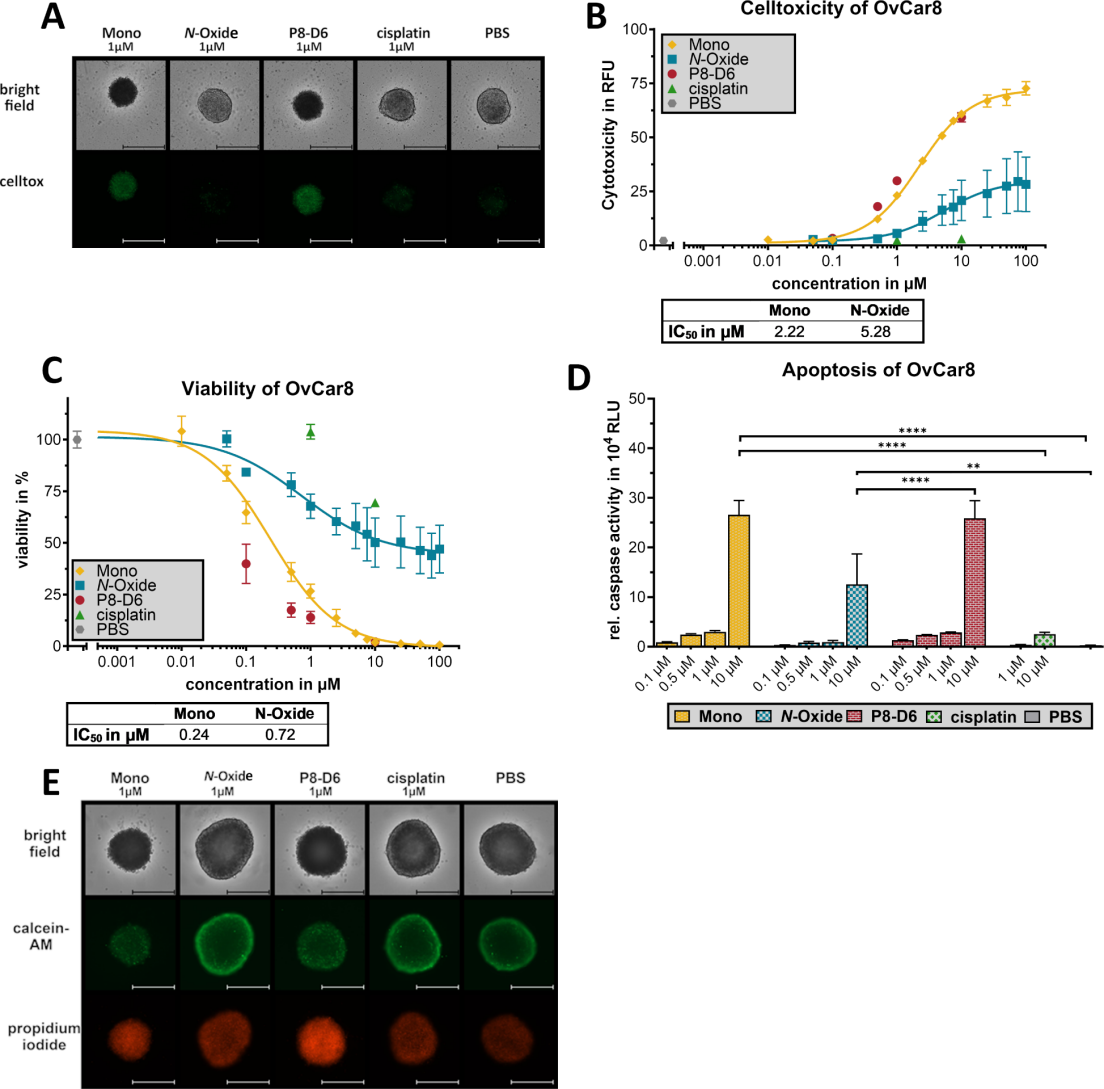
Anti-tumoral effect of P8-D6 mono and P8-D6 *N*-oxide in ovarian cancer 3D spheroids. OvCar8 were treated with P8-D6 mono, P8-D6 *N-*oxide, P8-D6, cisplatin, and PBS. During treatment, the cell toxicity was measured by fluorescence microscope using CellTox Green (A, B), scale bars, 500 μm. The fluorescence signals after 48 h of treatment were quantified (relative fluorescence units RFU) and shown in an IC_50_ curve (B). After 48 h of treatment, the viability (C) and caspase activity (D) were measured. The IC_50_ value of P8-D6 mono and P8-D6 *N-*oxide was calculated using the viability data (C). OvCar8 spheroids were stained after the growth and treatment phase with PI (red), calcein-AM (green), and Hoechst 33342 (blue) and measured by microscopy, scale bars, 500 μm. Data are means ± SEM (*n* = 3). Data are normally distributed (D’Agostino and Pearson test), ordinary one-way ANOVA (Šídák’s multiple comparisons test); **p < 0.01, ****p < 0.0001.

After treatment, the supernatants of the cell cultures were collected in order to quantify metabolites by HPLC. In Figure 3 C, the total metabolism in % (as the sum of all detected metabolites as a percentage of the substrate) is shown in dependency of the respective treatment. Overall, P8-D6 is always the most metabolized substrate at ∼5%, followed by P8-D6 *N*-oxide at ∼2.6% and P8-D6 mono at ∼1%. Since all three cell lines behave similarly, Figure 3 D shows the detailed metabolism of OvCar8. In contrast to previous incubation with homogenized tissue (Figure 2), P8-D6 was mostly metabolized to P8-DO (2.05%) and to a lesser extent to P8-D6 mono and P8-D6 *N*-oxide. P8-D6 mono was converted to P8-DO and less extensively metabolized to P8-D6 and a new unknown metabolite (0.10%). Due to the increased polarity and, therefore, decreased HPLC retention time, a nitrone would be thinkable. Such reactions have been described in the literature.^40–42^ However, due to the very limited content of this metabolite, it could not be identified further. During incubation with P8-D6 *N*-oxide, P8-D6 features the major metabolite, while P8-D6 mono and P8-DO were formed to a lesser amount. The protein expression of Cyp3A4, mARC1 and 2, and FMO3 in the cell lines used was analyzed by Western blot (Figure 3 E).

Tumor complexity is represented more realistically in 3D spheroid models than in 2D monolayer models. To assess efficacy, viability, caspase, and cytotoxicity in OvCar8 spheroids were analyzed after metabolite treatment.^5,34,43^ CellTox Green staining indicated a noticeable increase in cell toxicity due to treatment with P8-D6 (IC_50_ 1.11 μM), P8-D6 mono (IC_50_ 2.22 μM), and P8-D6 *N*-oxide (IC_50_ 0.31 μM) (Figure 4 A,B). P8-D6 mono proved to be a strong apoptosis inducer in 3D spheroids and achieved an IC_50_ value of 0.24 μM (Figure 4C,D). Further cytotoxicity studies include staining with calcein AM/propidium iodide, which revealed notably more effective cytotoxicity of target cells and changes in membrane integrity in spheroids treated with P8-D6 and P8-D6 mono (Figure 4E).

The inhibitory characteristics of P8-D6 on the classic efflux transporters P-GP and BCRP (ABCG2) were evaluated (Figure 5). P8-D6 possesses a minor inhibitory effect on calcein efflux by both P-GP and BCRP.

**Figure 5:**
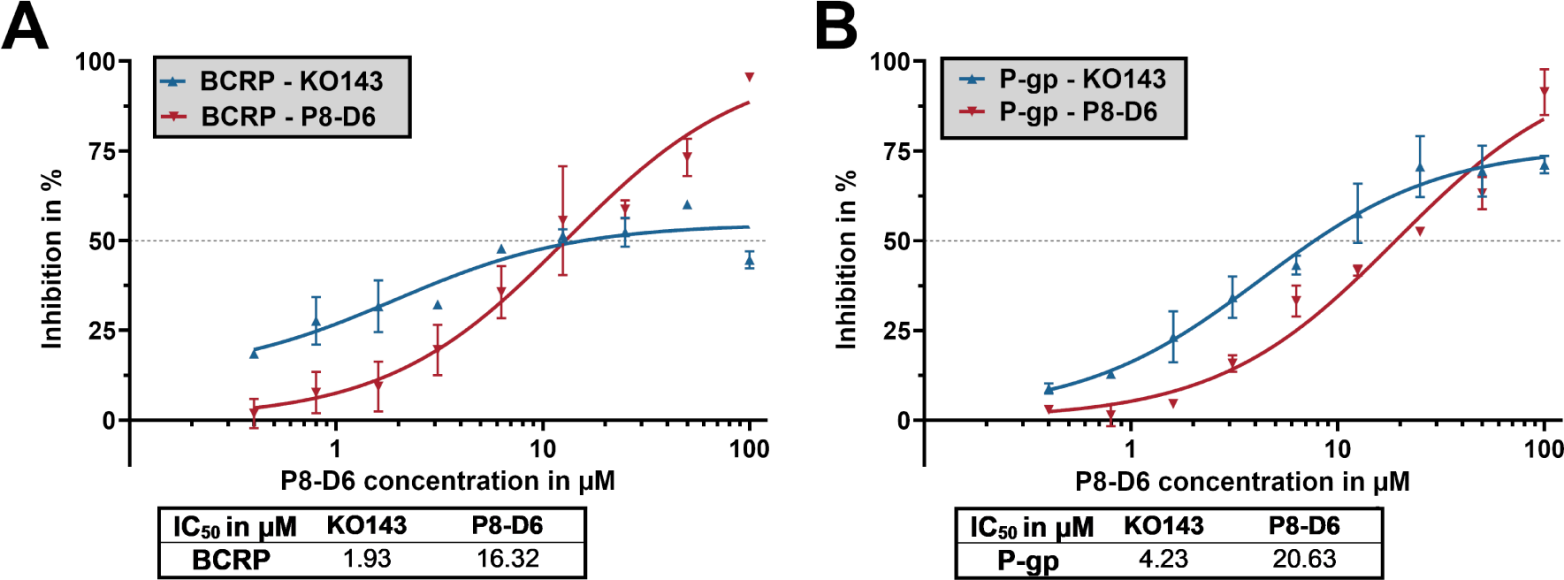
Inhibitory effect on the transporter proteins P-gp (A) and BCRP (B) by P8-D6 and KO-143 (positive control). IC_50_ values, defined as the concentration resulting in half-maximum inhibition, were determined. The inhibition was measured as intracellular accumulation of calcein-AM, expressed as mean ± SEM (*n* = 3).

### *In Vivo* Bioavailability, Distribution, and Metabolism

Animal treatment did not reveal limiting side effects. Organs were immunostained with antibodies to detect reactive oxygen species, DNA damage, and caspase levels, not showing abnormalities in the liver, lungs, spleen, kidneys, and colon (Figure 6E).

**Figure 6:**
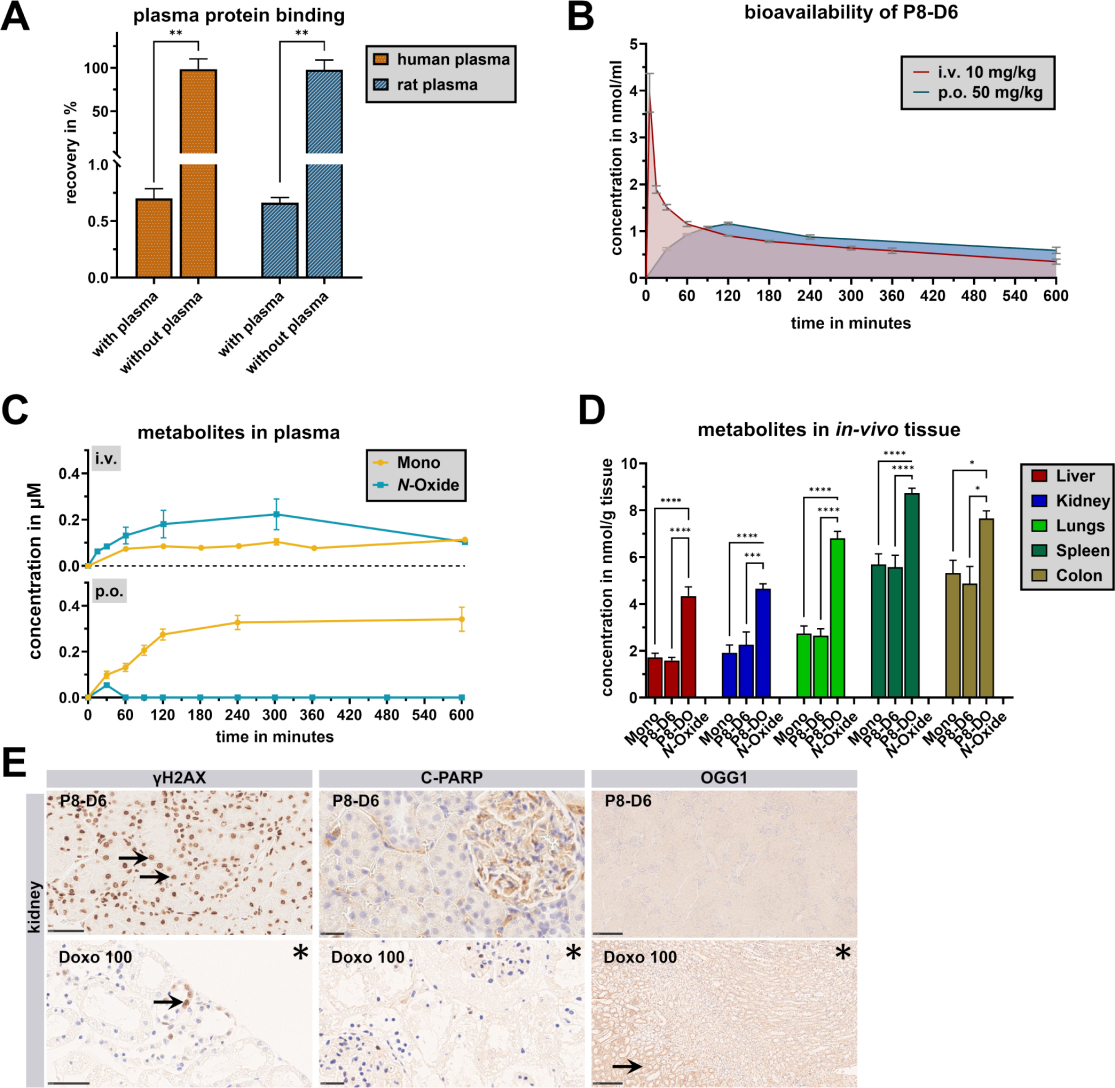
Estimation of P8-D6 plasma protein binding as the recovery of P8-D6 after incubation with and without human plasma and rat plasma (A). Data are means ± SEM (*n* = 5). Data are normally distributed (Kolmogorov–Smirnov test), Unpaired *t* test with Welch’s correction, two-tailed (SD not equal); **p < 0.01. *In vivo* plasma curves of P8-D6 after p.o. and i.v. administration (*n* = 6) (B). Detection of P8-D6 *in vivo* metabolites P8-D6 mono and P8-D6 *N*-oxide in plasma after p.o. and i.v. administration (C). Detection of P8-D6 and its metabolites in tissue after the *in vivo* experiment (D). The tissues liver, kidney, lung, spleen, and colon were examined immunobiologically for toxic changes. Data are means ± SEM (*n* ≥ 6). Data are normally distributed (Shapiro–Wilk test), one-way ANOVA (Tukey’s multiple comparisons test); *p < 0.05, **p < 0.01, ***p < 0.001, ****p < 0.0001. Immunohistochemical analysis of markers of tissue damage (E). Panel: kidney tissue. Doxo 100 = positive control. Asterisks: *ex vivo* analyses, scale bar: 50 μm (γH2AX), 25 μm (c-PARP), 250 μm (OGG1). Positive cells are marked with arrows.

To examine the effect of the P8-D6 plasma protein binding on the *in vivo* bioavailability, human and rat plasma was spiked *in vitro* and incubated with P8-D6. By using ultrafiltration to separate free P8-D6 from the protein-bound fraction, a plasma protein binding of 99% (Figure 6A) was estimated. However, neither human α-1-microglobulin (3.06%) nor human serum albumin (0.91%) is responsible for binding. According to initial findings, γ-globulins (94.59%), human transferrin (90.43%), LDL (87.99%), human α2-HS glycoprotein (85.29%), and human fibrinogen (78.89%) lead to binding of P8-D6.

### Bioavailability of P8-D6

To determine the absolute oral bioavailability, P8-D6 was applied *in vivo* to male Sprague–Dawley rats by p.o. (50 mg/kg body weight) and i.v. (10 mg/kg body weight). The plasma concentration–time profiles and pharmacokinetic parameters of P8-D6 after p.o. and i.v. administration are presented in Figure 6 B and Table 1.

After i.v. injection, the initial P8-D6 plasma concentration *c*_max/i.v._ (3.95 μM) was measured at 5 min followed by a significant decrease during the distribution phase within the first 60 min before reaching a *c*_min/i.v._ of 0.35 μM in the terminal elimination phase. After p.o. administration and an initial resorption phase, a *c*_max/p.o._ of 1.16 μM was observed after 120 min (*t*_max_). Subsequently, the elimination phase dominated, and over the next 480 min, the plasma concentration decreased until it reached its *c*_min/p.o._ at 0.59 μM. An absolute oral bioavailability of 21.5% was determined for P8-D6 (Table 2).

**Table 2:** Pharmacokinetic Parameters of P8-D6 after p.o. or i.v. Administration to Sprague–Dawley Rats

	P8-D6	
Adm. route	i.v.	p.o.
Dose in mg/kg BW	10.0	50.0
AUC_0–10 h_ in μM*min	449.3	481.7
Bioavailability in %	100%	21.5%
*c*_max_ in μM	3.95 ± 0.42	1.16 (measured), 1.25 (calculated)
*c*_min_ in μM	0.35 ± 0.05	0.59 ± 0.06
*t*_max_ in min	0 (at injection)	120 (measured), 105.9 (calculated)
Macro rate constants in min^–1^	α = 0.073 ß = 0.002	*k*_e_ = 0.0013 *k*_a_ = 0.031
Initial *t*_1/2_ in min	9.50	–
Terminal *t*_1/2_ in min	347.05	518.3
*V*_d_	2.06 l (9.36 L/kg)	2.96 l (13.46 L/kg)
*V*_c_	0.89 l (4.06 L/kg)	–
*V*_ss_	2.95 l (13.42 L/kg)	–
CL in L/h	0.38	0.24

Area under the curve from 0 to 10 h (AUC_0–10 h_). Maximum and minimum plasma concentrations are expressed as mean ± SEM, and *t*_max_ is given as mean (*n* = 6). All underlying equations are shown in the methods section. A body weight of 0.220 kg ± 0.015 kg was taken into account for calculations. *V*_d_ = volume of distribution, *V*_c_ = central volume of distribution, and *V*_ss_ = volume of distribution during the steady state.

Compartment models were used to calculate the pharmacokinetic parameters and plasma curves.

Since a pure drug solution of P8-D6 was administered in this study, liberation (release from the dosage form) could be neglected; therefore, first-order kinetics were assumed. Due to the log P of 2.9 (log D_7.4_ = 3.7), a relevant distribution and thus a two-compartment model were assumed.^30^ Based on our *in vivo* results and assumptions, P8-D6 is first homogeneously distributed in the central compartment after application before being transferred to the peripheral compartment. Elimination occurs via the central compartment. The substance transport into, out of, and between the different compartments is characterized by the macro rate constants.

Starting with the i.v. administration, a two-compartment pharmacokinetic model was chosen that considers the biexponential decay of a fast initial first-order distribution and a slow terminal first-order elimination phase (supplied as Figure S4A,C). By this method, a fictitious initial P8-D6 plasma concentration *c*_0/i.v._ of 4.43 μM at the time of injection was calculated (A (3.27 μM) + B (1.16 μM)). This fictitious plasma concentration corresponds to ∼1% (3.95 μmol in 8.8 mL^44^ of the theoretical plasma concentration and matches the plasma protein binding of P8-D6 of about 99% (Figure 6 A). Based on the dose and *c*_0_, a central *V*_c_ of 0.891 L was calculated. Since the macro rate constant for distribution is considerably greater (37-fold) than the one for elimination, the elimination is the rate-determining step according to first-order kinetics.

To cope with the overlapping curves of absorption and elimination after p.o. administration, compartmental models are used (supplied as Figure S4B,D). However, since only two to three data points exist (depending on the definitive start time of the elimination) during the elimination and distribution phase (120–600 min), no differentiation between these two phases can be described by our data (as shown by the residual method in Figure S4B). Therefore, a one-compartment model according to the Bateman function with both first-order invasion and elimination rates was assumed for the p.o. administration of P8-D6 (supplied as Figure S4D).^45^ Thus, a plasma half-life of 518.3 min was calculated. A fictitious initial plasma concentration, *c*_0/p.o._ = 2.21 μM, was calculated.

### Identification of Metabolites in Rats

Another part of this *in vivo* study was to identify *in vivo* metabolites and determine the tissue distribution of P8-D6 (study preparation scheme supplied as Figure S5). Interestingly, in plasma, no P8-DO was detected. After p.o. administration of P8-D6, the highest plasma concentration of metabolites was observed with P8-D6 mono at *c*_max/p.o._ 0.341 μM after 600 min (Figure 6D). P8-D6 *N*-oxide reached *c*_max/p.o._ of 0.054 μM at 30 min, and all following results were below the limit of quantification (LoQ). P8-D6 *N*-oxide proved to be the preferred plasma metabolite after i.v. administration of P8-D6, with a *c*_max/i.v._ of 0.228 μM after 300 min. P8-D6 mono had a lower concentration, with a *c*_max/i.v._ of 0.114 μM after 600 min.

The detected concentrations of P8-D6 and its metabolites in tissue material (spleen, liver, lungs, kidney, and colon) are shown in Figure 6D. In tissue, no P8-D6 *N*-oxide was observed at all, while P8-DO was considered to be the major tissue derivative, accounting for about 50% of the total proportion of all derivatives. P8-D6 and P8-D6 mono each constituted about 20–30% of the detected derivatives. The extent of metabolism was, to a certain degree, organ-dependent (spleen > colon > lungs > kidney > liver). The highest concentration of P8-DO was found in the spleen at 8.7 nmol/g and the lowest in the liver at 4.5 nmol/g.

Immunohistochemical analyses of γH2AX (marker for DNA damage) showed diffuse positivity in the P8-D8-treated tissue and the positive control of the kidney tissue. Immunohistochemical analysis of C-PARP (marker of apoptosis) detected no significant difference between the positive control, negative control, and P8-D6-treated sample. OGG1, a marker for oxidative DNA damage produced by reactive oxygen species, was expressed weakly in the background and cytoplasm of the negative control samples of kidney tissue. The positive control sample showed marked positivity in the cytoplasm of the tubular system of the kidney. The P8-D6-treated kidney tissue did not significantly differ from the negative control (Figure 6E). No specific immunohistochemical reactions could be observed for the other organs.

### *In Vivo* Elimination

To investigate the elimination of P8-D6 and its phase I and phase II metabolites, urine and feces samples were collected over the course of 24 h and examined.

To examine possible *in vivo* phase II metabolites, an enzymatic hydrolysis with glucuronidase/sulfatase from *Helix pomatia* was performed with both urine and feces samples. By comparing the P8-D6 recovery before and after incubation, a P8-D6-glucuronide was identified in both urine (+15.47% P8-D6 after incubation) and feces (+41.57% P8-D6) (Figure 7C,D). No sulfate phase II metabolites were observed.

**Figure 7:**
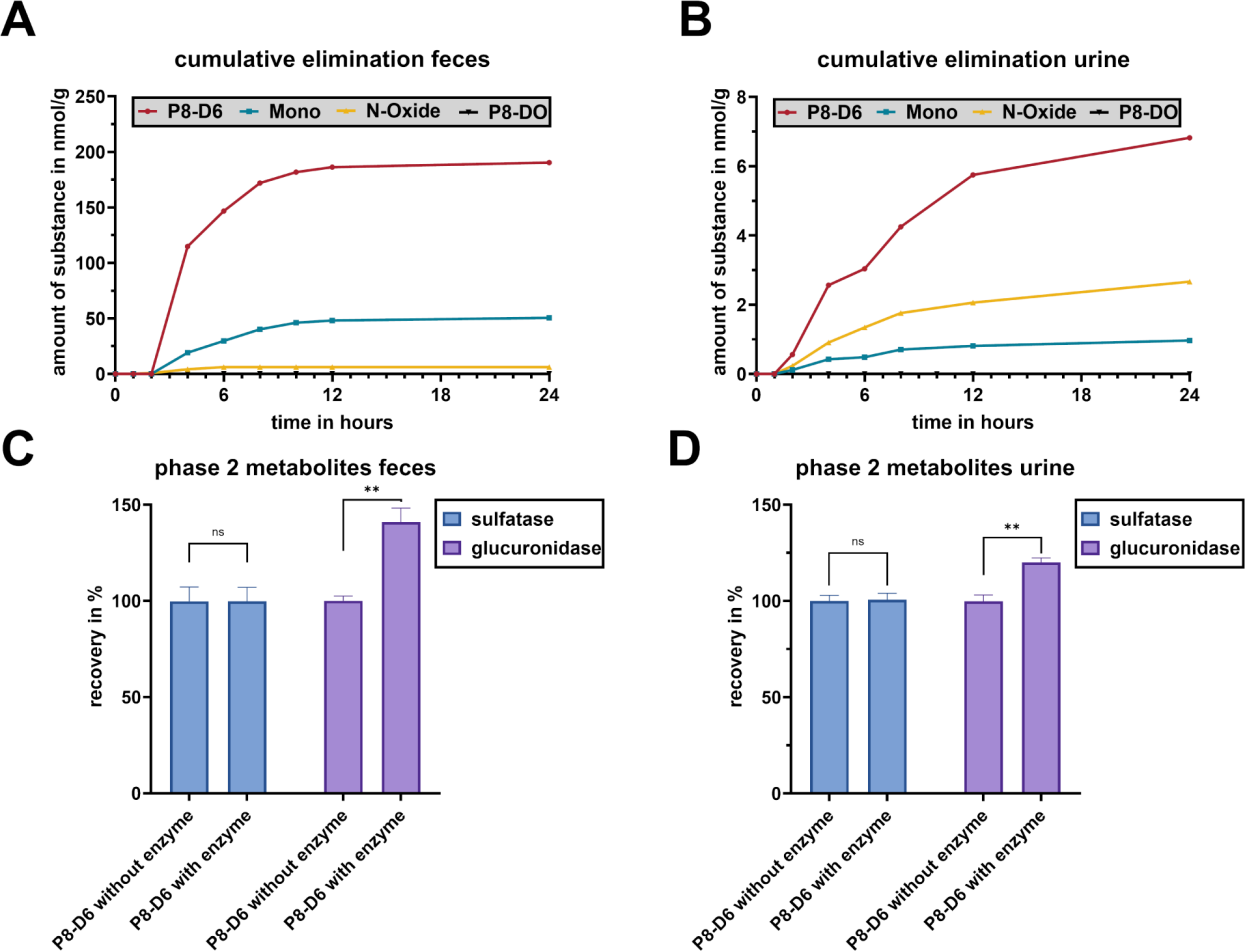
Cumulative elimination of P8-D6 (as the sum of P8-D6 and its glucuronide) and its phase I-metabolites via feces (A) and urine (B) over 24 h. Detection of phase II metabolites in feces (C) and urine (D). Data are means ± SEM (*n* = 3). Data are normally distributed (Shapiro–Wilk test), unpaired *t* test with Welch’s correction, two-tailed (SD not equal); **p < 0.01, one-way ANOVA; **p < 0.01 ns nonsignificant.

The results of the cumulative excretion via urine and feces are shown in Figure 7A,B. Among the known phase I metabolites, only P8-D6 mono and P8-D6 *N*-oxide were observed, while P8-DO was absent. In both matrices, the combined concentration of P8-D6 and its glucuronide always exceeded its metabolites. In feces, this difference was 3.7-fold between P8-D6 and P8-D6 mono and 31-fold regarding P8-D6 *N*-oxide after 24 h of cumulative observation. P8-D6 *N*-oxide was detected in the feces after 2 h at a concentration of 2.90 nmol/g, but it was only detectable up to 8 h. Contrary to feces, P8-D6 *N*-oxide is the most dominant phase I metabolite in urine. The renal elimination of P8-D6 *N*-oxide again began after 2 h. In total, 2.61% of the administered P8-D6 has been eliminated either as P8-D6 or its phase I and phase II metabolites during the first 24 h.

## Discussion

In preclinical studies, P8-D6 was a highly effective inducer of apoptosis in tumor cells by inhibiting both topoisomerase I and II through the formation of a covalent DNA-topoisomerase complex.^5,31–34^ While the simultaneous inhibition of both topoisomerases has great potential in oncology and can reduce tumor resistance, no dual topoisomerase I and II inhibitor is currently approved for cancer therapy due to severe side effects.^10,17,18,46–51^ High efficacy with low side effect potential makes P8-D6 an interesting drug candidate for the treatment of cancer.^5,32–34^

### ***In Vitro*** **Metabolism**

In the *in vitro* biotransformation study, the metabolism of P8-D6 in different species, organs, and subcellular fractions was investigated, and three metabolites (P8-DO, P8-D6 mono, P8-D6 *N*-oxide) in the low percentage range were detected. Subcellular fractions from different species were used as a proxy for the *in vivo* pharmacokinetic studies in rats and further pharmacodynamic studies in mice. Pig livers contain cytochrome P450 enzymes that are similar to those in humans, so their metabolic and conversion processes are similar but more active. This allows only limited predictions, as this animal material often has a higher metabolic activity than humans.^52–54^

The highest metabolic activity was obtained in microsomal fractions, which was to be expected since the most relevant phase I metabolic enzymes (FMOs and CYP enzymes) are membrane-associated enzymes. The relatively increased formation of P8-D6 *N*-oxide in the human PNS fraction can be explained by an increased expression of FMO3 in obesity and insulin resistance.^55,56^ The S9 fraction and PNS, which contain some additional metabolic enzymes, did not reveal any other metabolites.

When comparing the mixed-gender S9 fraction to the female-only S9 fraction, no significant differences regarding the extent of metabolism were found. Gender-specific differences in the expression of metabolic enzymes have been described in the literature, as CYP3A4 and FMO3 are generally more highly expressed in females than in males,^57–61^ but this was not significant for this study. Additionally, drug metabolism in females is strongly influenced by the menstrual cycle, menopause, and pregnancy. For instance, the activity of FMO3 is decreased during menstruation.^36,62^

Based on the detected phase I metabolites, the responsible metabolic enzymes were identified using recombinant enzymes. FMO3 exclusively catalyzed the formation of P8-D6 *N*-oxide, while P8-D6 mono was converted by CYP3A4, 2D6, 2B6, and 2C19. Human FMOs usually oxygenate soft nucleophiles, including the *N*-oxygenation of primary, secondary, and tertiary amines.^36^ CYP3A4 and 2D6 are the principal enzymes responsible for *N*-demethylation in human liver microsomes.^63,64^ All relevant CYP enzymes (mainly 3A4, 2D6, 2B6, and 2C9) for the formation of P8-D6 mono and P8-DO are subject to genetic polymorphism, with specific activities ranging from a poor metabolizer to an ultrarapid metabolizer.^65–67^ In the present study, human subcellular liver fractions from mostly caucasian origin were used.

Since valid *in silico* algorithms for predicting drug metabolism are established, a computer model (way2drug.com) was used for comparison. It predicted possible metabolic reactions and highlighted the affected atoms and involved enzymes (Figure 8).^68–70^ According to these results, a *N*-oxidation/*N*-oxygenation of the nitrogen on the side chain is the most probable metabolite (*p* = 0.8, P8-D6 *N*-oxide), followed by both a *N*-glucuronidation (*p* = 0.7) and a *N*-dealkylation of the same nitrogen (*p* = 0.65, P8-D6 mono). Indeed, the three most probable metabolites were detected both *in vitro* and *in vivo*, while P8-DO was not predicted. According to predictions, P8-D6 mono is catalyzed by CYP3A4 (*p* = 0.9) and 2D6, 2C19, 2C9, and 1A2 (*p* = 0.8). That perfectly matches the results of this study. However, the formation of P8-D6 *N*-oxide was predicted by CYP2C9 (*p* = 0.9), but *in vitro* only human FMO3 catalyzed this reaction, although *N*-oxidations by CYP enzymes are described in the literature.^71–73^ *N-*glucuronide catalyzed by UGT (*p* = 0.35) was predicted for biotransformation in phase II and was recovered in *in vivo* samples of urine and feces.

**Figure 8:**
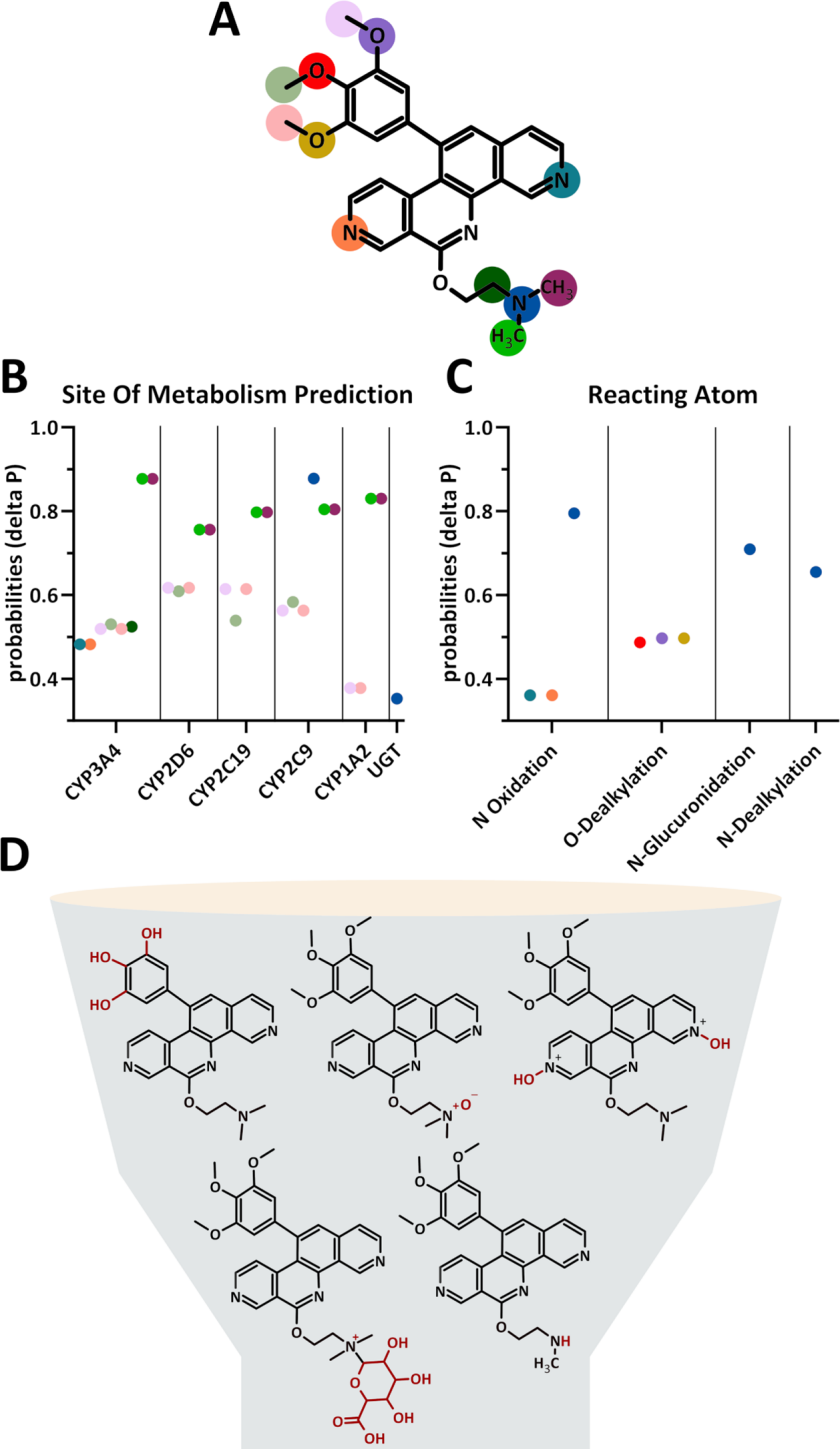
*In silico* prediction for drug metabolism by way2drug.com. Results for the *in silico* site of metabolism prediction for possible metabolic reactions for P8-D6. Graphical representation with color coding metabolic sites (A) indicating the probability of a CYP and UGT-mediated reaction (B) and the probability of specific chemical reactions (C). This results in predicted metabolites (D).

During *in vitro* incubations, species-specific and organ-specific differences regarding the extent of metabolism were observed. For instance, pigs featured the highest extent of metabolism followed by mice, rats, and humans in descending order. Besides the liver, lungs and kidneys seem to play an important role in the metabolism of P8-D6. These differences in the formation of P8-D6 *N*-oxide are mostly attributed to the species-specific and organ-specific expression of different FMO isoforms.^36,74,75^ In the human liver, FMO3 is the most frequent isoform. FMO1 is expressed in the kidneys, FMO2 and FMO4 are unspecific in many tissues, and FMO5 is in the duodenum and liver.^36,75,76^ Human expression of the several FMO isoforms differs from other species in extent and catalytic activity and therefore explains the differences in the formation of P8-D6 *N*-oxide observed in this study.^36,74,77–81^ As for pigs, especially high catalytic activities have been described in the literature, explaining the highest extent of metabolism among all examined tissues, subcellular fractions, and species.^78,79,82^

CYP3A4, 2D6, 2B6, and 2C19 enzymes are mostly expressed in the human liver and gastrointestinal tract.^76,83,84^ Once again, differences in the formation of P8-D6 mono and P8-DO are explained by the organ-specific constitution of species-specific analogues of human CYP isoforms, which show different levels of expression and catalytic activity.^52,85–87^ There is also the possibility that the P8-D6 *N*-oxide formed is subsequently reduced again by CYP enzymes or other metabolic complexes such as mARC (mitochondrial amidoxime reducing component).^36,88–90^ However, details of the metabolic pathways are not part of this study, as the mechanisms of CYP, FMO, and *N*-glucuronidation have already been described in detail in the literature.^36,77,91–97^

To correlate this result to other drugs, *in vitro* studies in human liver microsomes indicate that topotecan is metabolized to a *N*-demethylated metabolite. The mean ratio of metabolite to topotecan was approximately 3%, with P8-D6 to P8-D6 *N*-oxide metabolites at approximately 1%.^98^ Olaparib, which was approved for the treatment of ovarian cancer, is metabolized by CYP3A4, forming three major metabolites at a rate of 8–12%, and inhibits several enzymes and drug transporters, leading to a risk for drug–drug interactions.^99,100^

In the cell culture, P8-D6 shows the least metabolic stability, followed by P8-D6 *N*-oxide and P8-D6 mono. During drug design and development, the *in vivo* pharmacokinetic of a substance is often majorly influenced by the *in vitro* metabolic stability, which ideally accounts for the metabolic clearance. Therefore, initial target doses and dosing intervals can at least be partly derived from the metabolic stability.^101^ In addition to the high metabolic stability of P8-D6 mono, pharmacological effects were also observed in this study. In the “NCI-60 DTP Human Tumor Cell Lines Screening,” the GI_50_ value of P8-D6 mono was 37 nM compared to P8-D6’s 49 nM.^5^ *In vitro* pharmacodynamic data of P8-D6 monoconfirmed a similarly outstanding anti-tumor effect as for P8-D6. Although the efficacy of P8-D6 *N*-oxide is reduced 3-fold compared to P8-D6 mono in 3D spheroids, it is still 18-fold increased compared to cisplatin (13.51 μM). Thus, the main metabolites of P8-D6 are also effective against ovarian cancer and other entities. Since P8-D6 mono is highly effective and features a secondary amine at the side chain, several chemical modifications are possible to improve efficacy, target, and improve bioavailability through prodrug concepts.^102^ Altogether, this makes P8-D6 mono an interesting candidate for further drug development.

### *In Vitro* CYP Inhibition

Drug–drug interactions caused by the comedication of multiple drugs can result in altered exposure of the affected drug and raise safety or efficacy concerns. Since CYP enzymes are responsible for the biotransformation of many drugs, CYP inhibition by drugs poses a potential pharmacokinetic risk for newly developed drugs.^103–105^ Due to the low clinical relevance, possible interactions with FMO3 were not investigated. In fact, most studies that report possible drug–drug interactions base their observations on interindividual polymorphism of FMO3.^106–109^ For all investigated CYPs besides 2B6, an inhibition could be observed. However, since all IC_50_ values were well above 70 μM, no clinical significance of these results is expected.^110^

### *In Vivo* Bioavailability, Distribution, Metabolism, and Elimination

In this *in vivo* study with Sprague–Dawley rats, we determined the bioavailability of P8-D6 after p.o. (50 mg/kg BW) and i.v. (10 mg/kg BW) administration, the metabolism, and the elimination of P8-D6 and its metabolites in urine and feces. Although both administered doses exceeded the maximum tolerated dose of 5 mg/kg BW in the mice, no signs of toxicity have been observed.^30^ Although immunohistochemistry showed the marked expression of γH2AX, a marker of DNA damage in kidney tissue, the role of immunohistochemistry in determining a cytotoxic effect is limited. For the other DNA damage markers, c-PARP and OGG1 immunohistochemistry showed only a few positive cells in the control samples and sometimes scattered weakly positive cells in the P8-D6-treated samples. Based on these results, immunohistochemical analyses of these markers alone might not be a reliable method to account for specific tissue damage.

### Bioavailability

To determine bioavailability, P8-D6 and its metabolites were detected in plasma after both p.o. and i.v. administration, plasma curves were recorded, and several pharmacokinetic parameters were calculated. A fictitious plasma concentration of 1% of the theoretical concentration after i.v. administration perfectly corresponds to the estimated plasma protein binding of 99%. Additionally, the plasma concentration curve corresponds to the typical curve progression with a rapid distribution phase and a long phase of terminal elimination. After p.o. administration, the curve presents a Bateman kinetic with a plasma maximum after 120 min and about 50% remaining plasma concentration at the end of the experiment.

Due to the high volume of distribution and the biphasic decay in plasma concentration after the i.v. administration, the kinetics were best described by a two-compartment model (supplied as Figure S4A,C). Although the two-compartment model fits better, a one-compartment model was used due to the lack of plasma samples after p.o. administration during the terminal elimination phase (supplied as Figure S4B,D). In order to be able to compare both forms of application and since modeling such as PBPK requires more different pharmacokinetic values, the compartmental model was used here. Thus, two different clearances and plasma half-lives were observed during this study (CL_p.o._ 3.96 mL/min and CL_i.v._ 6.34 mL/min, 8.6 h (p.o.) and 5.8 h (i.v.)). For reference, imipramine has an average plasma half-life of 20 h during the terminal elimination phase.^111,112^ In addition to the *in vitro* metabolic stability, the *in vivo* metabolic stability of P8-D6 was determined in this study by using the plasma half-life, which is much more decisive for the pharmacokinetic profile and accumulation in the body than the *in vitro* results. Regarding the metabolites, no plasma half-lives were estimated since the metabolites were continuously formed and eliminated during the *in vivo* and *in vitro* experiments. Therefore, to identify the exact plasma half-lives of the metabolites, *in vivo* experiments with the metabolites instead of P8-D6 would be required. However, during this early stage of development and considering the scope and title of this manuscript, such experiments will be covered in future articles.

In addition to Lipinski’s “rule of five”, which P8-D6 fulfills (log *P* = 2.9, log D7.4 = 3.7),^30^ bioavailability is influenced by physicochemical and physiological factors like salt form of the drug, dosage form, drug complexation, food intake, and interaction with transporters.^113,114^ An oral bioavailability of 21.5% for P8-D6 was estimated in rats according to the area under the curves (AUC) of i.v. and p.o. application. For an estimation of the oral bioavailability based on the AUC_last_ (chapter Data Analysis), this is sufficient at this early stage of the project. However, future experiments should take this into account and enable the determination of the AUC_inf_ by a longer duration or by radioactive labeling. The predicted bioavailability score (by SwissADME) for P8-D6 was 0.55.^115^ In comparison, orally administered topotecan had a bioavailability of 35%.^116^ Therefore, P8-D6 is considered to be sufficiently orally absorbable.^117^

When comparing the pharmacokinetic parameters of P8-D6 with currently approved topoisomerase inhibitors, the plasma protein binding of P8-D6 (>99%) exceeds that of other drugs. However, as described in the literature, a high PPB does not necessarily limit a drug’s efficacy.^118–120^ Due to the high plasma protein binding and volume of distribution, some sort of drug depot might develop, leading to a high persistence and accumulation of the drug in the body. Targeted transport of P8-D6 to the tumor cells could also be a solution. Regarding clearance and plasma half-life, P8-D6 is well-positioned in the middle of the range among the approved topoisomerase inhibitors (Table 3). A p.o. administration of P8-D6 would allow patients to be treated at home and improve their quality of life. Since this study only examined bioavailability in rats, a determination in humans will be part of future studies. Clinical trials will detect whether P8-D6 achieves precise and repeatable plasma concentrations and whether p.o. or intravenous administration is suitable for individualized patient therapy.

**Table 3:** Pharmacokinetic Parameters of P8-D6 and Several Approved Selective Topoisomerase I or II Inhibitors^a^

Compound	Oral bioavailability in %	Protein binding in %	*V*_ss_ in L/m^2^	CL in L/(h m^2^)	*t*_1/2_ in h
P8-D6	21.5	>99	82.69	6.65 (p.o.)– 10.66 (i.v.)	5.7 (i.v.), 8.6 (p.o.)
topotecan^22,126,127,142,143^	32–44	7–35	43 (25–75), 130	14–34	3.1–4
doxorubicin^144–147^	5	50–85	809–1214	12–34	17.3–26
etoposide^22,123–125^	50 (40–75)	94–95	7.1 (5–17)	1–2	6.4
irinotecan^126–128^	14.1 (<10–20)	65	136–150	15	7.9–17
lurtotecan^126,127^	11.3 ± 5.2	90	190	60	3.7

a All values are expressed as mean (±SEM) and/or range. As for doxorubicin, the non-liposomal dosage form was taken into account for better comparability. A total body surface of 0.0357 m^2^ was taken into account for calculations.^148^

The clinical relevance of plasma protein binding is the subject of controversial discussions. The importance of the extent of plasma protein binding of a drug results from the fact that only the unbound molecule is responsible for its efficacy and that high plasma protein binding can reduce this.^118,120,121^ Nevertheless, there are several examples, such as the topoisomerase inhibitors etoposide (94–95%), lurtotecan (90%), or the EGFR inhibitor gefitinib (>97%), which have been approved for cancer therapy despite high PPBs.^22,122–128^ Another clinically relevant interaction potential of high plasma protein binding is the protein binding shift. Displacement by other drugs, altered concentrations of plasma proteins during stress situations, inflammations, and surgery can cause significant changes in the pharmacodynamics and toxicity of the drug. *In vitro* plasma protein binding models are static, as there is no elimination and distribution. However, rapid distribution, as with P8-D6, can often lead to increased penetration into the target tissue *in vivo* and, therefore, greater efficacy. However, interaction with comedications must be checked again separately for their pharmacokinetics. In addition, for a displacement to become relevant, the total concentration of both drugs must be in the magnitude of the affected plasma protein concentration, which would be way above therapeutic concentrations. Furthermore, possible drug–drug interactions due to displacement are much less likely for drugs which are eliminated mainly hepatic (with a low extraction ratio) rather than renally.^121,129–141^

### Metabolism and Distribution

All *in vitro* metabolites of P8-D6 have been detected *in vivo*. Due to the high volume of distribution, an accumulation of P8-D6 and its metabolites in tissues/organs was expected. The relative differences in the extent of metabolism are most likely explained by the previously discussed organ-specific constitution of metabolic enzymes. The increased concentration of P8-DO could be due to its low water solubility and high lipophilicity. The detection of the highest concentration of P8-D6, P8-DO, and P8-D6 mono in the spleen and colon may indicate an increased affinity for these tissues. Since only P8-D6 and P8-D6 mono were detected in feces, all three metabolites are most likely being formulated by colon tissue enzymes themselves instead of gut bacteria.

After i.v. administration, P8-D6 *N*-oxide was the main metabolite in plasma, while after oral administration, P8-D6 mono featured the highest concentration. Low levels of P8-D6 *N*-oxide after oral administration can be explained by both elimination and a reverse reaction to P8-D6 by reduction of P8-D6 *N*-oxide. *In vivo* reductions of *N*-oxides (e.g., by mARC) have been described in the literature.^36,88–90^ However, due to the general species and organ-specific composition of the metabolic isozymes, the results of *in vivo* metabolism cannot be directly transferred to human metabolism.^36,52^

### Elimination

In the past, the prediction of the renal and hepatic elimination of drugs has been successfully derived from animal experiments.^53^ In this study, both P8-D6 and its metabolites, P8-D6 *N*-oxide and P8-D6 mono, are eliminated by feces and urine, while P8-D6 always features the highest concentration of all three derivatives. P8-DO was completely absent. P8-D6 mono is the main metabolite in feces, and P8-D6 *N*-oxide is the main metabolite in urine. Furthermore, the predicted P8-D6 glucuronide was identified as a phase II metabolite in feces and urine. After 24 h, a total of 2.61% of the administered P8-D6 was eliminated by both urine and feces as either P8-D6 or its phase I or phase II metabolite. Most of the administered P8-D6 was accumulated in the rats due to the high plasma protein binding and distribution in tissue and therefore was unavailable for direct elimination; still, almost all free P8-D6 was eliminated from the plasma. In fact, this observation is quite similar to the elimination kinetics of doxorubicin, where at higher distribution volumes, 25–45% of the drug is eliminated after 7 days.^149^ Since phase II metabolites like P8-D6 glucuronide feature the possibility of an enterohepatic circulation where gut bacterial glucuronidase hydrolyzes the glucuronide and the product (P8-D6) is subsequently absorbed again, this additionally may explain the long duration in the organism and the low total cumulative elimination of 2.61% at the end of the experiment (12.13% when accounting for oral bioavailability).^150^

### Conclusion

P8-D6 is a novel dual topoisomerase I/II inhibitor and a promising drug candidate for cancer therapy. Previous *in vitro* studies proved its outstanding efficacy in gynecologic cancer and multiple myeloma compared to current standard therapy.^5,32,33^

In this *in vitro* and *in vivo* study, P8-D6 demonstrated very promising pharmacokinetic parameters in terms of oral bioavailability, distribution, and excretion. Only very small amounts of P8-D6 are enzymatically converted into its main metabolites (P8-D6 mono, P8-D6 *N*-oxide, and P8-DO), two of which are very effective. Most importantly, P8-D6 mono demonstrates high potency while offering higher metabolic stability and structural optimization opportunities for pharmacokinetics and bioavailability due to the secondary amine function. Therefore, P8-D6 mono may also be a good candidate for further development.^102^ In addition, further developments in drug formulation to improve the bioavailability would be useful. Since the transfer of *in vitro* and *in vivo* pharmacokinetics from animal models to the human organism is only possible within limits, simulations in advanced pharmacokinetic computer models are advised before clinical trials. Pharmacodynamic data from in vivo studies are important for this modeling. In summary, P8-D6 and especially its metabolite P8-D6 mono are very promising drug candidates for transfer to clinical trials in the foreseeable future.

## Experimental/Material and Methods

### Chemicals

All compounds are >95% pure by HPLC. P8-D6 was synthesized as recently described.^30^ P8-D6 *N*-oxide and P8-D6 mono were synthesized as described in section 2.3. All synthesized substances were tested for purity immediately before use. Acetonitrile (Honeywell Deutschland Holding GmbH, Offenbach, Germany) and methanol (J. T. Baker, Deventer, The Netherlands) were both of HPLC grade quality. Cisplatin was obtained from the UKSH (University Medical Center Schleswig-Holstein, Campus Kiel) dispensary.

Water was double-distilled and filtered through a 0.45 μm Pall hydrophilic polypropylene membrane filter (Cytiva Life Sciences , formerly Pall Life Sciences , Dreieich, Germany).

All other chemicals were purchased from either Sigma-Aldrich (Merck KGaA, Darmstadt, Germany) or Carl Roth (Karlsruhe, Germany).

### Tissue/Enzymes

Both commercial and internally processed tissue material and subcellular fractions were used in this study (supplied as Table S1). Internally processed materials originate from the following species and were homogenized as described below. Rat tissue was obtained from Sprague–Dawley rats (Janvier Laboratories, Le Genest-Saint-Isle, France). Mouse tissue was collected from mice of strain C57BL/6J (The Jackson Laboratory, Bar Harbor, Maine, USA). Pig liver (*Sus domesticus*) was provided by a local butcher. For human material, a pool of liver tissue from 81 donors was provided by the biobank of the clinic for visceral surgery at the UKSH Kiel (University Medical Center Schleswig-Holstein, Campus Kiel, ethic approval (A110/99; P2N2024-021)). The organs were immediately shock-frozen in liquid nitrogen upon collection, transported on dry ice, and finally stored at −80 °C.

### Homogenization

The organs were thawed on ice, gently squeezed to remove the remaining blood, and weighed. Afterward, a piece of 150–300 mg was cut off with a scalpel, divided into small parts, and homogenized with 1500 μL of ice-cold buffer (containing 0.25 M saccharose, 50 mM KCl, 1 mM EDTA, 10 mM potassium dihydrogen phosphate, and 1 mM dithiothreitol at pH 7.4) for about 5 min using a Potter S homogenizer (Sartorius, Goettingen, Germany) until no particles were visually detected. This homogenized tissue was centrifuged at 600 g for 10 min to obtain the postnuclear supernatant. Protein content was determined using a bicinchoninic acid (BCA) protein assay kit (Pierce, Rockford, USA) according to the manufacturer’s protocol.

### *In Vitro* Biotransformation, CYP Inhibition, and Plasma Protein Binding

For all phase I metabolism studies (except cell cultures), a 0.3 mL reaction mixture consisting of 1 mM substrate (P8-D6, P8-D6 mono, P8-D6 *N*-oxide), 1 mM NADPH as a cofactor, 0.67 mg/mL protein from homogenized tissue or subcellular fractions, respectively (except recombinant enzymes, where 0.5 mg/mL was used), and PBS buffer (pH 7.4) was used. All experiments were performed in biological triplicates. The FDA Guidance for Industry^39^ highlights 7 CYP isoenzymes (1A2, 2B6, 2C8, 2C9, 2C19, 2D6, and 3A4/5) which routinely should be tested for metabolism during drug development. Table S1 presents an overview of the tissue material and species as well as the CYP enzymes used in this study. All reactions were carried out at 37 °C in a shaking water bath for 150 min before they were stopped with 0.3 mL of an ice-cold mixture of acetonitrile/methanol (1:1). The suspension was vortexed for 30 s and centrifuged at 15000 g for 5 min, and finally, 10 μL of the supernatant was injected into the HPLC. Control samples were prepared with buffer instead of either protein/tissue, cofactor, or substrate.

To examine a possible *in vitro* CYP inhibition, the following CYP enzymes and marker substrates were investigated according to the FDA:^37,39^ phenacetin (CYP1A2), bupropion (CYP2B6), amodiaquine (CYP2C8), diclofenac (CYP2C9), amitriptyline (CYP2C19), dextromethorphan (CYP2D6), testosterone (CYP3A4), and nifedipine (CYP3A4). Incubation was performed in a 0.2 mL reaction mix consisting of PBS buffer (pH 7.4), 1 mM marker substrate, 1 mM NADPH, and either 0.5 mg/mL recombinant CYP enzyme or human liver microsomes. P8-D6 was added for a final concentration of 0.5 μM, 1 μM, 5 μM, 10 μM, 25 μM, 50 μM, 100 μM, 500 μM, 1000 μM, and (in some cases) 5000 μM. Sample preparation was performed according to *in vitro* biotransformation.

To examine the extent of plasma protein binding, 150 μL of blank samples of human plasma (1 mM) and rat plasma (1 mM), human albumin (1 mM, #A1653, Merck KGaA, Darmstadt); human α1-acid glycoprotein (1 mM; #G9885, Merck KGaA, Darmstadt); human α2-HS-glycoprotein (0.1 mM; #G0516, Merck KGaA, Darmstadt); human transferrin (0.2 mM; #T8158, Merck KGaA, Darmstadt); human fibrinogen (0.2 mM; #F3879, Merck KGaA, Darmstadt); LDL (0.2 mM; #); and human γ-globulin (1 mM; #G4386, Merck KGaA, Darmstadt) were spiked with P8-D6 in the same final concentration as the protein and incubated in a shaking water bath at 37 °C for 120 min. Afterward, the unbound fraction of P8-D6 was separated by ultrafiltration (Vivaspin 500 10 000 MWCO filter). The sample preparation was performed according to plasma samples. Blank plasma samples served as the control samples.

### *In Vitro* Transporter Inhibition Study

A potential interaction of P8-D6 with the export protein P-GP (ABCB1) was evaluated in MDCK (P-GP overexpression) and MDCKII (BCRP overexpression) cells. P-GP interaction was determined by the measurement of calcein accumulation within the cells according to Bauer et al.^151^ Calcein-acetoxymethylester (calcein-AM) is a nonfluorescent, lipophilic and therefore highly cell-permeable ester. Once inside the cell, the ester bonds are rapidly cleaved by nonspecific esterases, generating highly fluorescent calcein, which is trapped inside the cell because of its hydrophilic nature. Because calcein-AM is a substrate for P-GP, inhibition of the transporter decreases efflux, which results in a higher intracellular fluorescence signal. Cells were washed 3 times with 37 °C Krebs–Ringer buffer and subsequently incubated with increasing concentrations of the test compound for 15 min at 37 °C. Calcein-AM (MoBiTec, Göttingen, FRG) was added to a final concentration of 1 μM and incubated for 30 min at 37 °C. Afterward, the cells were immediately washed 3 times with ice-cold Krebs–Ringer buffer and lysed with 1% Triton X-100. Fluorescence was measured using a Fluoroskan Ascent plate reader (Labsystems, Helsinki, FIN) with an excitation wavelength of 485 nm and an emission wavelength of 520 nm. Each determination was repeated 3 times in 2 experiments to ensure reproducibility. All fluorescence values were corrected by subtracting the background fluorescence. The increase in cellular fluorescence caused by the test compound was referred to the fluorescence of the control (100%). KO-143 served as the positive control test compound and PBS as the negative control.

### Chromatographic Conditions

The setup for HPLC consisted of a reversed-phase separation principle. Therefore, a Shimadzu Nexera HPLC system consisting of an SCL-40 system controller, an LC-40D XR Solvent Delivery Pump, an SIL-40C XR Autosampler, a CTO-40C Column Oven, and an SPD-M40 PDA Detector equipped with a Shim-pack GIST-HP C18 column (3 μm; 100 mm × 4.6 mm) and a Shim-pack GIST-HP (G) C18 guard column (3 μm; 10 mm × 3.0 mm) was used for the determination and quantification of the metabolites (Software: Shimadzu LabSolutions (version 5.106)). The column temperature was kept at 40 °C while the flow rate was 0.9 mL/min. The mobile phase consisted of an isocratic mixture of 28% acetonitrile and 72% 100 mM ammonium acetate (pH 4.5). Each sample analysis had a runtime of 20 min. Peaks were detected at 260 nm and quantified by linear regression using standard curves (*R* ^2^ > 0.999). Data analysis was performed according to chapter Data Analysis. Calibration samples were prepared by spiking the respective matrix with the same stock solution of P8-D6 used in the respective study. Sample preparation was performed according to each study. At least five calibration levels were prepared with the expected P8-D6 concentration of the respective study at about 50% of the calibrated range.

To identify new metabolites, the method was transferred to an LC-MS system and validated with injections of P8-D6. The chromatographic system consisted of an Agilent Series 1260 Infinity II HPLC system (Agilent Technologies, Waldbronn, Germany), with a 1260 μ-Degasser, a 1260 binary pump, a 1260 TCC column oven with the temperature set to 40 °C, and a 1260 HiP ALS autosampler with a 1290 thermostat set to 4 °C. We used the same guard column, separation column, and mobile phase as mentioned above to identify all peaks by their retention time. Mass spectra were recorded from *m*/*z* 70 to *m*/*z* 1000 using a Bruker Amazon SL mass spectrometer with ion trap and electrospray ionization (Bruker Daltonik, Bremen, Germany) with positive polarity. Voltages were set to capillary −4200 V and end plate −500 V, nebulizer at 8.0 psi, and 4.2 L/min nitrogen at 250 °C as drying gas. Evaluation of the data was carried out with the Bruker Data Analysis 4.0 software package. After the initial identification of the metabolites, these were postsynthesized. These postsynthesized substances were used to revalidate the LC-MS method.

### Synthesis of Metabolites

All identified metabolites were chemically synthesized. These compounds served as HPLC standards and were subjected to further analysis in cell culture experiments. For spectroscopic data and HPLC purity, see Supporting Information.

P8-DO is a direct precursor of P8-D6 and is synthesized as described previously.^30^

P8-D6 mono: 200 mg (0.484 mmol) of P8-DO and 379.9 mg (1.45 mmol) of triphenylphosphine were added to a flask and dissolved in 200 mL freshly dried THF under a nitrogen stream. Afterward, 245 μL (1.45 mmol) of *N*-Boc *N*-methyl aminoethanol and 285 μL (1.45 mmol) of DIAD were slowly added consecutively at 0 °C. The reaction mixture was stirred for 72 h under argon, during which the temperature was allowed to increase to room temperature. Then, the total volume of the reaction mixture was reduced to 10**–**20 mL using a rotary evaporator and dissolved in 200 mL of DCM. Two M HCl in ether was added to that mixture until no more crystals of P8-D6 mono hydrochloride precipitated. The product was obtained by filtration. Finally, P8-D6 mono was purified by preparative HPLC (Interchim PuriFlash 4250, Uptisphere Strategy C18-HQ 10 μm 250 × 21.2 mm prep-LC column, 23 mL/min flow, detection at 254 nm, linear gradient from 20% ACN 80% 0.1 AcOH to 50% ACN at 12 min) and obtained by freeze-drying. Yield: 98.1 mg (0.208 mmol, 43%) of a yellowish crystalline solid.

**P8-D6 *N*-oxide:** 60 mg (0.124 mmol) of the free base of P8-D6 was dissolved in methanol. Afterward, 3 mL of aqueous H_2_O_2_ (15%) was added to the reaction mixture which was consequently heated to 65 °C and stirred for 2 h. Unreacted traces of H_2_O_2_ were removed by evaporation, and the product was purified by preparative HPLC. Finally, the product was obtained by freeze-drying. Purification was carried out by the method mentioned above. Yield: 55.9 mg (0.112 mmol, 90%) of a yellow-whitish crystalline solid.

### Cell Culture

The human ovarian cancer cell lines OvCar8, Igrov-1, and A2780, purchased from ATCC, were maintained in RPMI1640 medium, including l-glutamine supplemented with 10% fetal bovine serum and 60 IU/mL penicillin–streptomycin. Cells were grown at 37 °C and 5% CO_2_ in a humidified incubator and subcultivated at a confluency of 70–80%. Cell line authenticity was checked by STR profiling as described previously,^152^ and mycoplasma contamination was routinely investigated using MycoAlert (Lonza).

### 2D Viability Apoptosis Assay

For the measurements of viability and apoptosis, cells of approximately 8000/well were seeded in a Corning No. 3903 plate and treated with the corresponding drugs for 48 h. After treatment, apoptosis and cell viability assays were performed using an ApoLive-Glo Multiplex Assay Kit (Promega #G6410) according to the manufacturer’s instructions. Fluorescence and luminescence units were measured by using a microplate multimode reader (Spark, Tecan). Relative caspase activity was calculated as caspase activity divided by viability (normalized to control). With viability data, dose–response curves were plotted, and inhibitory concentration 50% (IC_50_) values were calculated. All experiments were performed in biological triplicates. PBS was used as the negative control, and cisplatin and P8-D6 were used as the positive controls.

### 3D Cytotoxicity, Viability, and Apoptosis Assay

OvCar8 cells (8000/well) were seeded into an Ultra-Low-Attachment plate (Corning #4520) and grown for 96 h. Then, spheroids were treated with the drugs for 48 h. Cell cytotoxicity was assessed using the CellTox Green Cytotoxicity Assay (Promega #G8731) 48 h after treatment with NYONE (SYNENTEC, 485Ex/520Em). Filters: BFEx/GreenEm (530/43 nm); BlueEx (475/28 nm)/GreenEm (530/43 nm). Viability and apoptosis were determined by RealTime-Glo (460Em) (Promega #G9711) and caspase-Glo 3/7 (565Em) (Promega #G8090) according to the manufacturer’s instructions using a microplate reader (Spark, Tecan). Relative caspase activity: caspase activity divided by the viability (normalized to control). For live-dead staining, cells were grown and treated as described above. Then, 80% of the medium was removed and replaced with propidium iodide (PI) (15 μM), calcein-AM (0.2 μM), and Hoechst 33342 (1.8 μM) in medium for 3 h and imaged by NYONE (SYNENTEC). Filters: BFEx/GreenEm­(530/43 nm); Hoechst 33342: UVEx (377/50 nm)/BlueEm (452/45 nm); calcein-AM: BlueEx (475/28 nm)/GreenEm (530/43 nm); propidium iodide: LimeEx­(562/40 nm)/RedEm­(628/32 nm). All experiments were performed in biological triplicates. PBS was used as the negative control, and cisplatin and P8-D6 as the positive control.

### Western Blots

Cells were harvested, protein contents were determined, and SDS-PAGE and Western Blot analysis were carried out as described previously.^34^ For analysis, membranes were incubated with primary antibodies at 4 °C overnight (anti-FMO3 1:10000 (Proteintech #17469-1-AP); anti-CYP3A4 1:10000 (Proteintech #182271-AP); anti-MOSC1/2 1:10000 (Sigma-Aldrich #ZRB2392)) and then washed with TBST. Further, the membranes were incubated with HRP-labeled goat antirabbit IgG 1:1000 (Cell Signaling #7074S). After washing with TBST, membranes were developed with ECL Substrate (Bio-Rad #1705061) or SuperSignalTM West Femto Maximum Sensitivity Substrate (Thermo Scientific #34096). The protein levels of HSP90 were used as loading controls. All experiments were performed in biological triplicates.

### *In Vivo* Study and Sample Preparation

Male Sprague*–*Dawley rats (6–7 weeks of age, body weight of 220 g ± 15 g) received a standard diet and water *ad libitum* under a controlled 12 h day/night light cycle at room temperature. The studies were approved by the Committee for Animal Protection in Baden-Württemberg, Germany (AZ 35-9185.81/G-223/21). The study followed the ARRIVE guidelines and EU regulations on animal research throughout the research process.

To investigate the *in vivo* bioavailability, metabolism, and elimination of P8-D6 after p.o. and i.v. application, two groups of rats were dosed with 50 mg/kg body weight orally (*n* = 6) and 10 mg/kg body weight (*n* = 6) intravenously. Effects of the diet were reduced by fasting the rats overnight prior to P8-D6 application. Blood samples were taken at 5, 15, 30, 60, 120, 180, 300, 360, and 600 min after i.v. application or 30, 60, 90, 120, 240, and 600 min after p.o. application. Immediately afterward, the plasma was collected by centrifugation at 2000 g for 10 min. The rats were housed in metabolic cages to collect the urine in specially attached containers. Urine and feces samples were collected every 2 h. The rats were euthanized by CO_2_ inhalation. The harvested organs (kidney, spleen, lung, liver, and colon) were weighed and submerged in liquid nitrogen. All collected samples were stored at −80 °C until analysis.

### Plasma Sample Preparation

50 μL of plasma was mixed with 100 μL of an ice-cold mixture of acetonitrile/methanol (2:1) and stirred in the vortex for 5 min. The suspension was centrifuged at 15000 g for 10 min, and 50 μL of the supernatant was injected into the HPLC (chapter Chromatographic Conditions). Calibration/control samples were prepared the same way with plasma samples collected from equal Sprague–Dawley rats from nonpharmacological studies. For calibration, the matrix was spiked with a defined concentration of the analyte. Final concentrations ranged from 0 μM to 5 μM. Triple determinations were performed and prepared on each respective day. LoD: 0.02 μM, LoQ: 0.05 μM (plasma).

### Urine and Feces Sample Preparation

Thirty mg of feces was added to 100 μL of a 0.15 M NaCl solution and stirred in the vortex for 30 s. Afterward, 200 μL of an ice-cold mixture of acetonitrile/methanol (2:1) was added before vortexing again for 30 s. Finally, the suspension was centrifuged at 15000 g for 5 min. 40 μL of the supernatant was injected into the HPLC. The samples from three mice per group were analyzed. Calibration/control samples were prepared the same way with fecal samples collected ahead of the *in vivo* study. For calibration, the matrix was spiked with a defined concentration of the analyte. Final concentrations ranged from 0 nmol/g to 200 nmol/g. Triple determinations were performed and prepared on each respective day. 50 μL of urine was mixed with 100 μL of an ice-cold mixture of acetonitrile/methanol (2:1) and stirred in the vortex for 5 min. The suspension was centrifuged at 15000 g for 5 min, and 40 μL of the supernatant was injected into the HPLC. Calibration/control samples were prepared the same way with urine samples collected ahead of the *in vivo* study. For calibration, the matrix was spiked with a defined concentration of the analyte. Final concentrations ranged from 0 to 10 μM. Triple determinations were performed and prepared on each respective day. LoD: 0.02 μM (urine), 0.22 nmol/g (feces); LoQ: 0.06 μM (urine), 0.72 nmol/g (feces). Additionally, to identify phase II metabolites, feces (30 mg) and urine (50 μL) from the *in vivo* study were incubated with arylsulfatase (at pH 6.2) and glucuronidase (at pH 4.5) from *Helix pomatia* in acetate buffer for 2 h in a 37 °C water bath. For reference, feces and urine without enzyme and 4-nitrophenylsulfate and 4-nitrophenylglucuronide were incubated as well. All experiments were performed in biological triplicates.

### Tissue Sample Preparation

100 μL of each tissue homogenate/PNS was mixed with 200 μL of an ice-cold mixture of acetonitrile/methanol (2:1) and stirred in the vortex for 5 min. The suspension was centrifuged at 20000 g for 10 min, and 50 μL of the supernatant was injected into the HPLC. The samples from six mice per group were analyzed. Calibration/control samples were prepared the same way with organ samples collected from equal Sprague–Dawley rats from nonpharmacological studies prepared according to chapter Tissue/Enzymes. For calibration, the matrix was spiked with a defined concentration of the analyte. Final concentrations ranged from 0 to 10 nmol/g. Triple determinations were performed and prepared on each respective day. LoD: 0.81 nmol/g (spleen), 0.33 nmol/g (liver), 0.48 nmol/g (lungs), 1.14 nmol/g (colon), 0.38 nmol/g (kidney); LoQ: 2.69 nmol/g (spleen), 1.09 nmol/g (liver), 1.59 nmol/g (lungs), 3.80 nmol/g (colon), 1.28 nmol/g (kidney).

### Histology and Immunohistochemistry

Tissues were fixed with 4% formaldehyde and dehydrated with an ethanol series before embedding in paraffin. Tissue sections were cut using a microtome; the tissue sections were deparaffinized and rehydrated in water. Hematoxylin and eosin (H and E) staining was carried out on a Tissue Tek Prisma Plus autostainer (SAKURA). For immunohistochemistry, sections were stained with antibodies directed against γH2AX (Cell Signaling #9718, dilution 1:200, DNA-damage), OGG1 (Proteintech #15125-1-AP, dilution 1:200, DNA-damage), and c-PARP (Abcam #ab32064, dilution 1:200, DNA-damage). Antigen retrieval was achieved with ER2 (EDTA-buffer Bond pH 9.0; 20 min). The immunoreaction was visualized with the Bond Polymer Refine Detection Kit (DS 9800; brown labeling; Novocastra; Leica Biosystems GmbH), resulting in a brown color and counterstained with hematoxylin. The IHC stainings were carried out on the autostainer BOND RX system (Leica Biosystems GmbH). The stained tissue sections were digitalized with a NanoZoomer S60 Digital slide scanner (Hamamatsu) at a 40× magnification objective and analyzed by board-certified pathologists. The samples from three mice were analyzed.

### Data Analysis

For data analysis and statistics, Microsoft Excel 2019 and GraphPad Prism (version 10.2, GraphPad Software Inc., San Diego, California), commercially available software, were used. All data are expressed either as mean ± SEM or ± SD. The area under the curve (AUC) was calculated by the trapezoidal method. Only data above the limit of quantification and within the working range up to the last measurement were considered for the calculation of the AUC (AUC_last_). For smoothing, all available data were used except for results which deviated more than 50% from the average, which were therefore excluded. This applies to both the i.v. and p.o. application of P8-D6. Gaussian normal distribution was assured by the D’Agostino and Pearson test, Shapiro–Wilk test, or Kolmogorov–Smirnov test. Data of two groups were analyzed using Student’s *t*-test. Student’s *t*-tests were performed according to an unpaired, parametric *t*-test with Welch’s correction, two-tailed (SD not equal) (95% CI, definition of significance: *p* < 0.05). For comparison of more than two variables/groups, an ordinary one-way ANOVA with Šídák’s multiple comparisons test or Tukey’s multiple comparisons test was chosen (95% CI, *p* < 0.05). To calculate IC_50_ values, a nonlinear regression model by the least-squares regression method (95% CI, α = 0.05) was chosen.

The *in vivo* compartment models were calculated as described in Supporting Information (3. Methods).

## Supplementary Material



## Notes

Figure S1: Inhibition of the CYP; Figure S2: growth in 60-tumor cell line panel by NCI; Figure S3: antitumor effect of P8-D6 mono and P8-D6 *N*-oxide in ovarian cancer 2D cell culture; Figure S4: method of residuals two-compartment model; Figure S5: preparation scheme; Table S1: general HPLC method validation; Table S2: method validation for the sample preparation of biological matrices; Table S3: overview of tissue and species for *in vitro* incubation; MS, HPLC and NMR spectra of P8-D6 mono, P8-D6 *N*-oxide, P8-DO, and P8-D6; list of enzymatic material; method of pharmacokinetic analysis of concentration–time data (PDF)

## Abbreviations

ACN: acetonitrile
AcOH: acetic acid
AUC: area under ther curve t1/2 biological half-life
BCRP: breast cancer resistance protein
BW: body weight
CI: confidence interval
CL: clearance
*c*_max_: maximum concentration
*c*_min_: minimum concentration
CO_2_: carbon dioxide
C-PARP: cleaved poly-ADP-ribose polymerase
DCM: dichloromethane
DIAD: diisopropyl azodicarboxylate
Doxo 100: doxorubicin 100 μM
FMO: flavin-containing monooxygenases
Gamma-H2AX/ γ-H2AX: phosphorylation of the histone variant H2AX
GI_50_: growth inhibition 50%
H_2_O_2_: hydrogen peroxide
H_2_SO_4_: sulfuric acid
H_3_PO_2_: hypophosphorous acid
HCl: hydrochloric acid
HRP: horseradish peroxidase
HSP: heat shock protein
i.v.: intravenous
KCl: potassium chloride
KO*t*Bu: potassium *tert*-butoxide
LoD: limit of detection
LoQ: limit of quantification
mARC: mitochondrial amidoxime-reducing component
MDCK: Madin Darby canine kidney
*N*: sample size
NaNO_2_: sodium nitrite
*N*-Boc: *N*-*tert*-butoxycarbonyl
OGG1: 8-oxoguanine DNA glycosylase-1
p.o.: per oral
PARP: poly ADP-ribose polymerase
Pd/C: palladium on carbon
pH: potential of hydrogen
PNS: postnuclear supernatant
RFU: relative fluorescence units
RLU: relative luminescence unit
SD: standard deviation
SDS-PAGE: sodium dodecyl sulfate polyacrylamide gel electrophoresis
SEM: standard error of the mean
STR: short tandem repeat
TBST: tris-buffered saline with Tween 20
*t*_max_: time-to-maximum
TPP: triphenylphosphine
UGT: UDP-glucuronosyltransferase
*V*_c_: central volume of distribution
*V*_d_: volume of distribution
*V*_ss_: volume of distribution during the steady state
