# Supplementary material for: In Vivo and In Vitro Pharmacokinetic Studies of a Dual Topoisomerase I/II Inhibitor
Source: ACS Pharmacol Transl Sci. 2025 Mar 12;8(4):1050–71. doi: 10.1021/acsptsci.4c00596 (PMC11997890; doi:10.1021/acsptsci.4c00596)
Supplement: Supplementary file 1 [file pt4c00596_si_001.pdf]

In vivo and *in vitro* pharmacokinetic studies of a dual topoisomerase I/II inhibitor

Jonas Hildebrandt[1], Dirk O. Bauerschlag[2,5], Gert Fricker[3], Ulrich Girreser[1], Björn Konukiewitz[4], Franziska Kellers[4], Nicolai Maass[2], Bernd Clement[1,*] and Inken Flörkemeier[1,2,*]

1 Christian-Albrechts-University Kiel, Pharmaceutical Institute, Department of Pharmaceutical and Medicinal Chemistry, 24118 Kiel, Germany; jhildebrandt@pharmazie.uni-kiel.de; bclement@pharmazie.uni-kiel.de; girreser@pharmazie.uni-kiel.de

2 Department of Gynaecology and Obstetrics, University and University Medical Center Schleswig-Holstein Campus Kiel, 24105 Kiel, Germany, inken.floerkemeier@uksh.de; Nicolai.Maass@uksh.de

3 Ruprecht-Karls University, Institute of Pharmacy and Molecular Biotechnology, 69120 Heidelberg, Germany, gert.fricker@uni-hd.de

4 Department of Pathology, University and University Medical Center Schleswig-Holstein Campus Kiel, 24105 Kiel, Germany, Bjoern.Konukiewitz@uksh.de; Franziska.Kellers@uksh.de

5 Department of Gynecology and Reproductive Medicine; Jena University Hospital, 07747 Jena, Germany, Dirk.Bauerschlag@med.uni-jena.de

* Correspondence

Content

1. Supporting Figures S2
2. Analytic/Chemistry S4
3. Methods S12
4. References S14

Figure S 1. Inhibition of the CYP S2
Figure S 2. Growth in 60-tumour cell line panel by NCI S3
Figure S 3. Anti-tumor effect of P8-D6 Mono and P8-D6 N-Oxide in ovarian cancer 2D cell culture. S3
Figure S 4. Method of residuals two-compartment model S4
Figure S 5. Preparation scheme S12

Table S 1: General HPLC method validation. S5
Table S 2: Method validation for the sample preparation of biological matrices. S5
Table S 3: Overview of tissue and species for in vitro incubation. S12

1. Supporting Figures

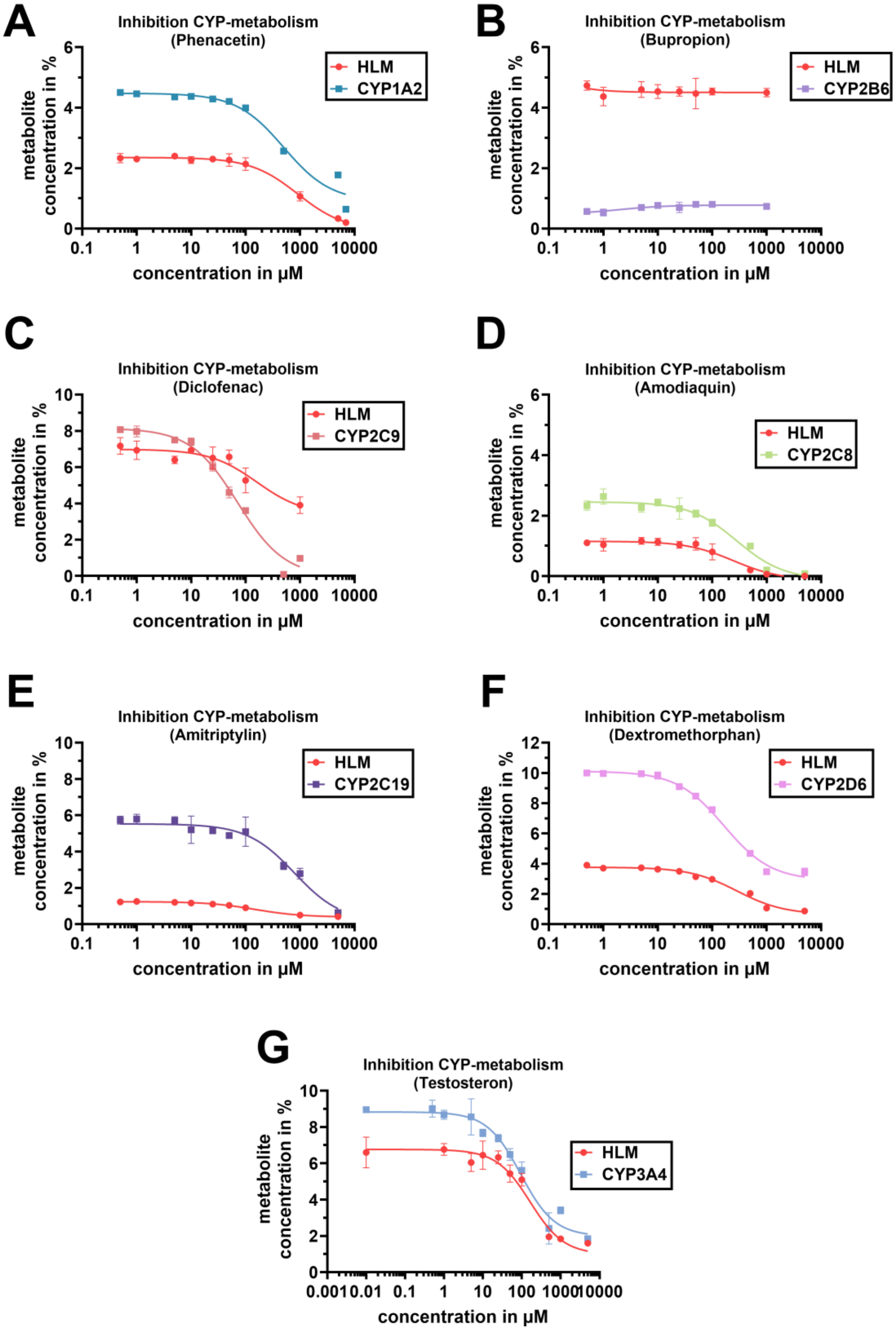

Figure S 1. Inhibition of the CYP

P8-D6 mediated Inhibition of the CYP in vitro marker reactions according to FDA compared to HLM (human liver microsomes). The investigated CYPs were 1A2 (A), 2B6 (B), 2C9 (C), 2C8 (D), 2C19 (E), 2D6 (F) and 3A4 (G). All results are expressed as mean ± SD (n = 3).

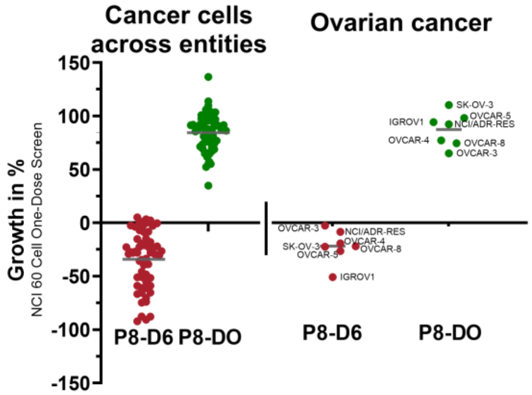

Figure S 2. Growth in 60-tumour cell line panel by NCI
NCI 60 Cell One-Dose Screen detecting tumor growth after treatment with P8-D6 and P8-DO in 60 cancer cell lines of different entities and in ovarian cancer cell lines.

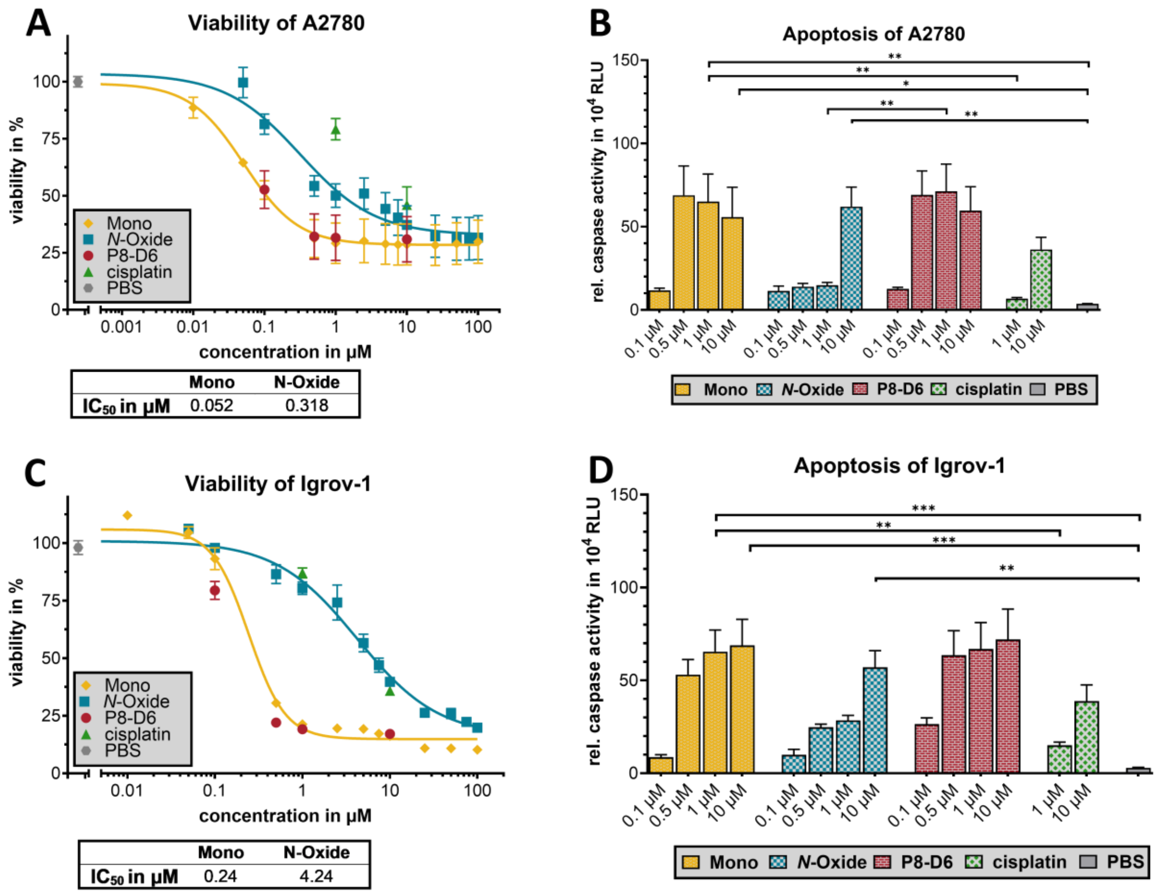

Figure S 3. Anti-tumor effect of P8-D6 Mono and P8-D6 N-Oxide in ovarian cancer 2D cell culture.
A2780 (A,B)and Igrov-1 (C,D) were treated for 48 hours with of P8-D6 Mono, P8-D6 *N*-Oxide, P8-D6, Cisplatin and PBS. Subsequently, the viability (A, C) and caspase activity (B,D) were measured. The IC50 value of P8-D6 Mono, P8-D6 *N*-Oxide was calculated using the viability data (A, C). Data are means + SEM (n = 3). Data are normally distributed (D'Agostino & Pearson test), one-way ANOVA (Šídák's multiple comparisons test); * ($p < 0.05$), ** ($p < 0.01$), *** ($p < 0.001$), **** ($p < 0.0001$).

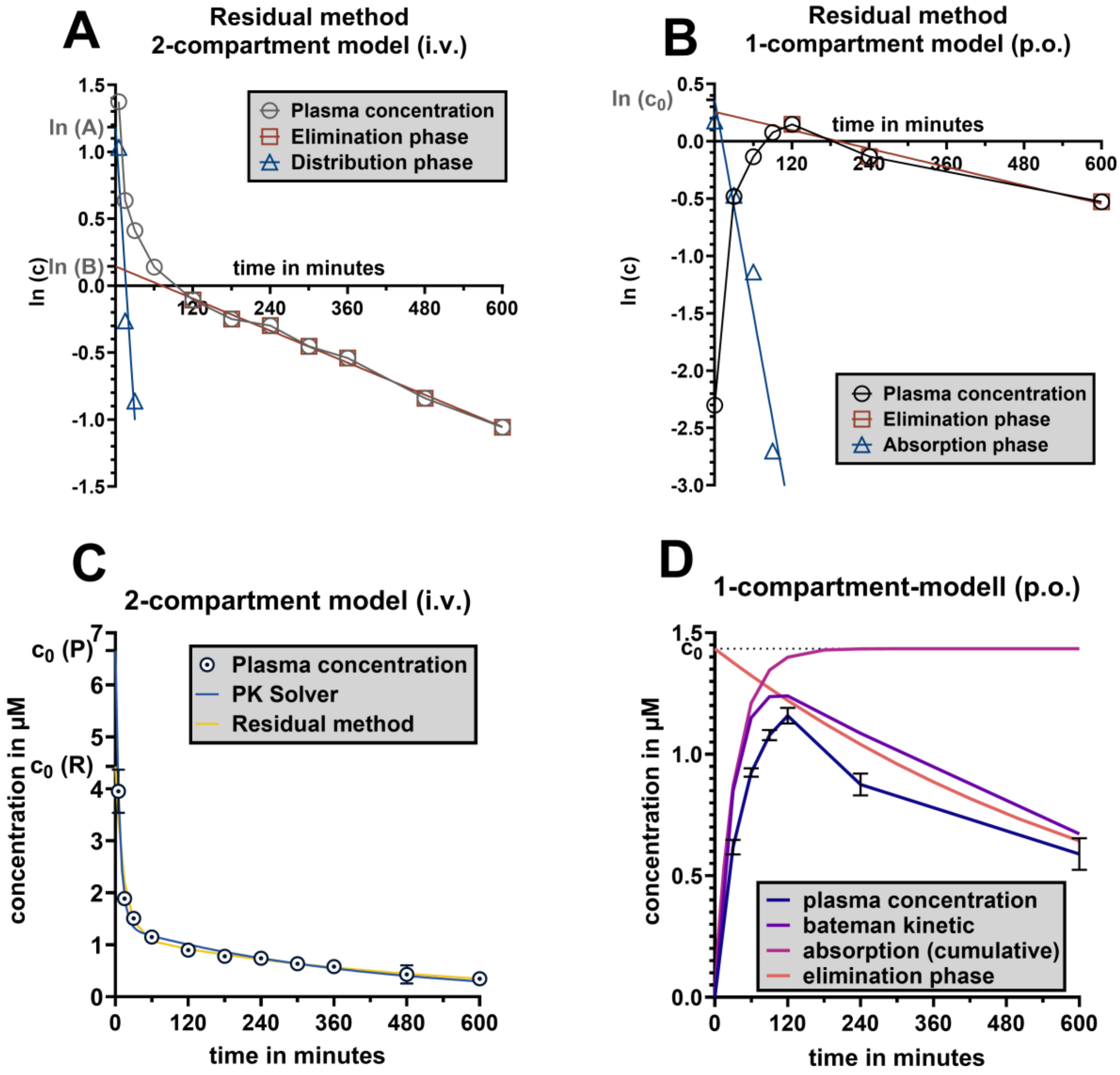

Figure S 4. Method of residuals two-compartment model

(A) for i.v. administration and one-compartment model (B) for p.o. administration as performed as described in the Methods chapter). Figure C and D show the *in vivo* observed and calculated plasma curves for each respective compartment model ("residual method" and "bateman kinetic"). PKSolver was used as a reference for method validation (C).[1] Figure D further presents the cumulative absorption as well as the remaining amount of drug which has to be eliminated.

2. Analytic/Chemistry

MS, IR, HPLC and NMR spectra

^{1}H (400 MHz) and ^{13}C (100 MHz) NMR spectra were recorded on a Bruker Avance III, 400, spectrometer at 298 K equipped with Bruker TopSpin 3.6.1 software. Chemical shifts (δ values) are expressed in ppm in relation to TMS as an internal standard. NMR Spectra were interpreted by first order analysis. Low resolution mass spectra were recorded using a Bruker Amazon SL system with LC coupling, electrospray ionization, in the positive mode. IR spectra were recorded on a Shimadzu IR Affinity-1S FTIR spectrometer equipped with MIRacle 10 single reflection ATR accessory. For HPLC-method see chapter 4.5.

HPLC method validation

The HPLC method has been validated according to chapter 2.2.46 of the Ph. Eur., <621> USP and the "Reviewer-Guidance" of the CDER („Center for Drug Evaluation and Research") of the FDA. The results are presented in the following tables.

Table S 1: General HPLC method validation.

Parameter	Requirement	Result
Reduced plate height (h)	< 3	2.844
Number of theoretical plates (N)	> 2000	10045
Asymmetry factor (A_s)	0.8 – 1.5	1.499
Tailing factor (T_F)	0.8 – 1.5	1.272
Capacity factor (k‘)	> 2	6.665
Resolution (R_s)	> 1.5	1.52

For HPLC-method see chapter 4.5.

Table S 2: Method validation for the sample preparation of biological matrices.

	Range (min – max)	Linearity (R^2)	Accuracy (%)	Precision (RSD %)	Recovery (% ± SD)	Limit of detection	Limit of quantification
Spleen (nmol/g)	2.69 – 149.18	0.99996	98.58	2.99	106.55 ± 2.61	0.81	2.69 *
Liver (nmol/g)	1.09 – 88.35	0.99999	99.79	2.41	96.70 ± 2.17	0.33	1.09 *
Lungs (nmol/g)	1.82 – 202.42	0.99996	100.39	1.75	103.68 ± 4.30	0.48	1.59
Colon (nmol/g)	3.80 – 133.77	0.99984	100.85	2.63	101.42 ± 2.07	1.14	3.80 *
Kidney (nmol/g)	1.29 – 29.65	0.99999	98.93	2.04	100.83 ± 4.89	0.38	1.28
Plasma (µM)	0.50 – 25	0.99982	99.05	2.39	72.68 ± 5.74	0.02	0.05
Urine (µM)	0.50 - 26	0.99990	97.93	1.96	102.94 ± 3.58	0.02	0.06
Feces (nmol/g)	23.21 – 1933.24	0.99926	99.50	2.21	97.17 ± 2.20	0.22	0.72

*For HPLC-method see chapter 4.5. For samples marked with an *, the limit of quantification has defined the lower limit of the working range.*

Spectrographic and analytical data

P8-D6 Mono (IUPAC: 6-(2-Methylaminoethoxy)-11-(3,4,5-trimethoxyphenyl)pyrido[3,4-c][1,9]phenanthroline hydrochloride. SMILES: CNCCOc1c2cnccc2c3c(cc4ccncc4c3n1)-c5cc(c(c(c5)OC)OC)OC)

Spectroscopic Data: ^{1}H NMR (400 MHz, DMSO + MeOH): δ/ppm = 2.70 (t, 3H, H-3”, ^{3}J = 5.2 Hz), 3.60 (m, 2H, H-2”), 3.76 (s, 6H, 2 x OCH3), 3.83 (s, 3H, 1 x OCH3), 5.28 (m, 2 H, H-1”), 6.88 (s, 2H, H-2‘ u. H-6‘), 7.36 (d, 1H, H-9, ^{3}J = 6.3 Hz), 8.28 (s, 1H, H-12), 8.56 (d, 1H, H-1, ^{3}J = 6.3 Hz), 8.75 (d, 1H, H-10, ^{3}J = 6.3 Hz), 8.94 (d, 1H, H-2, ^{3}J = 6.3 Hz), 9.57 (m, 2H, N-H2), 10.08 (s, 1H, H-7), 10.65 (s, 1H, H-4). ^{13}C NMR (100 MHz, DMSO + CDCl3): δ/ppm = 35.8, 49.7, 56.4 (2C), 60.8, 65.7, 106.3 (2C), 115.2, 116.6, 119.0, 120.2, 124.6, 126.2, 136.3, 137.8, 138.0, 139.5, 142.5, 143.6, 146.3, 148.3, 149.4, 150.0, 154.0 (2C), 159.5. IR (ATR): $\tilde{\nu}$/cm^{-1} = 3383, 1639, 1577, 1498, 1456, 1415, 1379, 1338, 1242, 1111, 995, 829. MS (ESI): m/z = 471.2 $[M+H]^{+}$, 414.1 $[P8\text{-}DO+H]^{+}$, 235.9 $[M+2H]^{++}$

HPLC purity: 99.24 %

HPLC

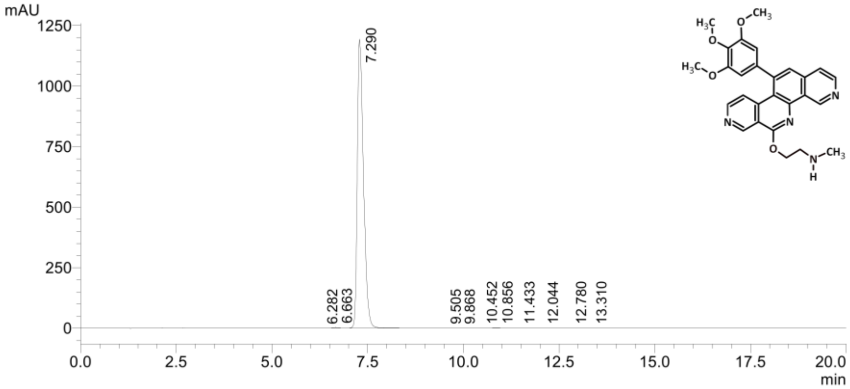

PDA Ch2 260nm

Peak#	Ret. Time	Area	Height
1	6.282	7496	410
2	6.663	33858	3303
3	7.290	13548871	1189619
4	9.505	5354	265
5	9.868	12081	669
6	10.452	9062	546
7	10.856	20058	1086
8	11.433	2935	164
9	12.044	5922	165
10	12.780	2901	104
11	13.310	3666	149
Total		13652203	1196480

LC-MS

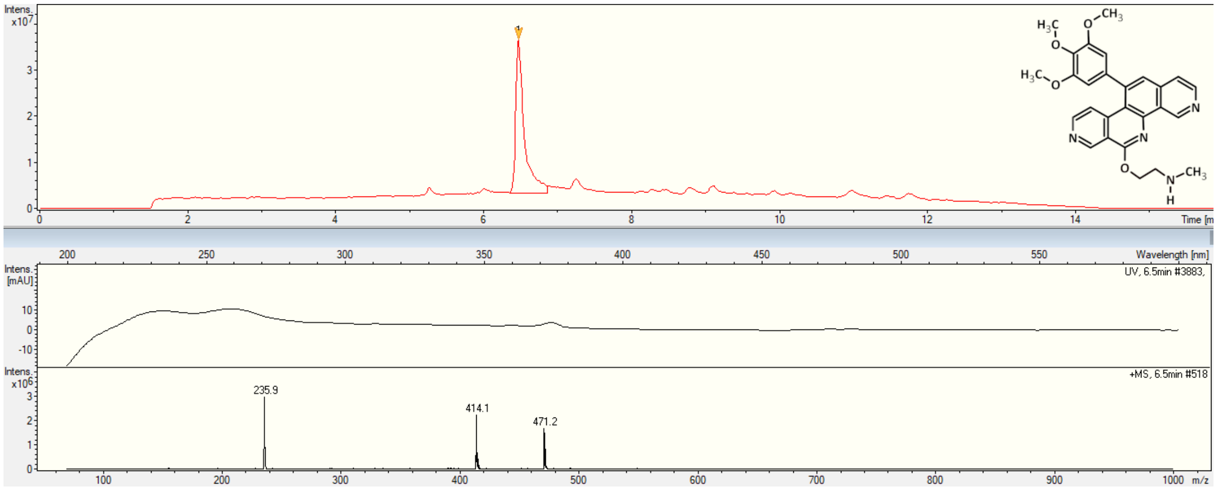

H-NMR

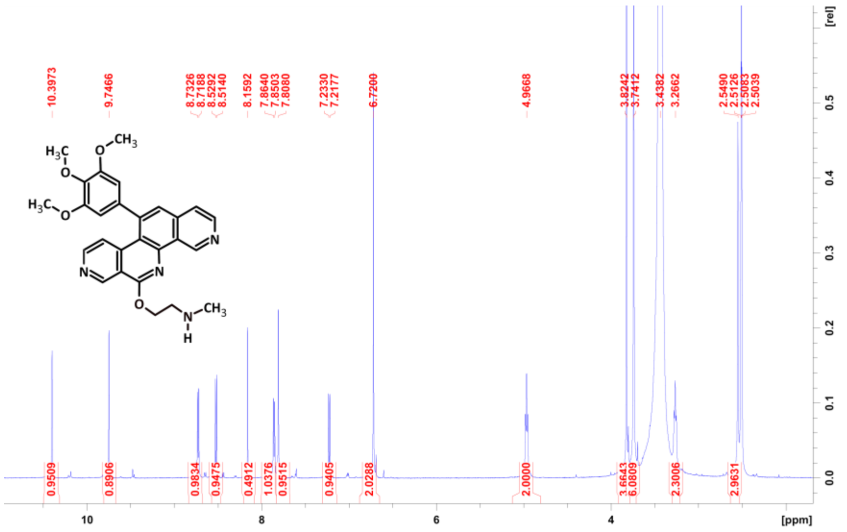

10.3973
9.7466
8.7326
8.7188
8.5292
8.5140
8.1592
7.8640
7.8503
7.8080
7.2330
7.2177
6.7200
4.9668
3.8242
3.7412
3.4382
3.2662
2.5490
2.5126
2.5083
2.5039
0.9509
0.8906
0.9834
0.9475
0.4912
1.0376
0.9515
0.9405
2.0288
2.0000
3.6643
6.0809
2.3006
2.9631
[ppm]
[rel]

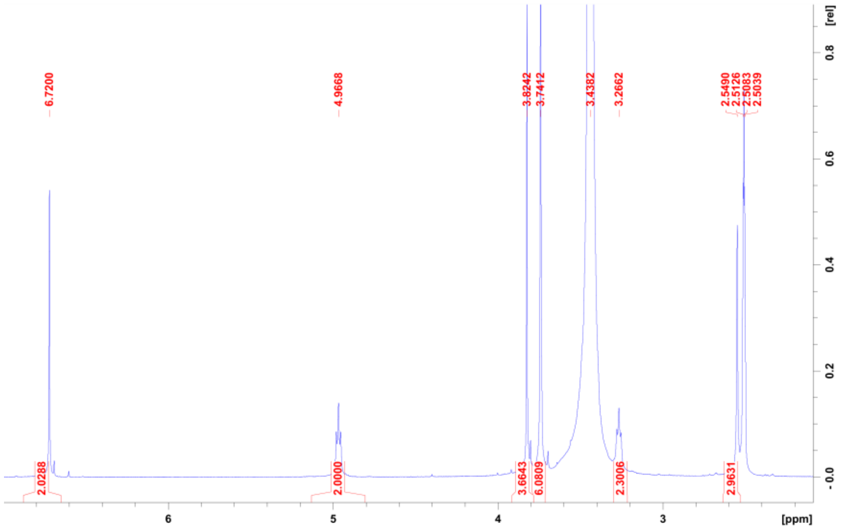

6.7200
4.9668
3.8242
3.7412
3.4382
3.2662
2.5490
2.5126
2.5083
2.5039
2.0288
2.0000
3.6643
6.0809
2.3006
2.9631
[ppm]
[rel]

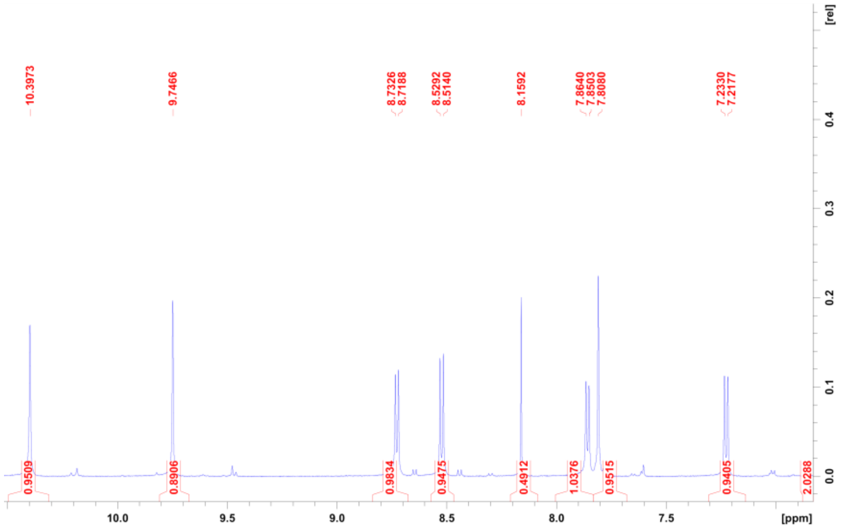

10.3973
9.7466
8.7326
8.7188
8.5292
8.5140
8.1592
7.8640
7.8503
7.8080
7.2330
7.2177
0.9509
0.8906
0.9834
0.9475
0.4912
1.0376
0.9515
0.9405
2.0288
[ppm]
[rel]

C-NMR

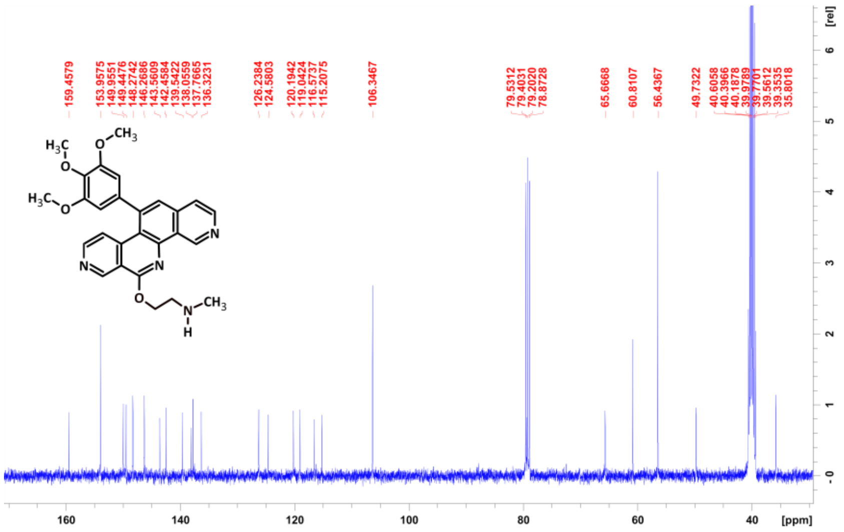

P8-D6 N-Oxide (IUPAC: 6-(2-Dimethylaminoethoxy)-11-(3,4,5-trimethoxyphenyl)pyrido[3,4-c][1,9]phenanthroline N-Oxide. SMILES:C[N@@+]([O])(CCOc1nc2c(c(cc3c2cncc3)c4cc(c(c(c4)OC)OC)OC)-c5c1cncc5)C)

Spectroscopic Data: ^{1}H NMR (400 MHz, DMSO + MeOH): δ/ppm = 3.31 (s, 6H, H-3" + H-4"), 3.73 (s, 6H, 2 x OCH3), 3.80 (s, 3H, 1 x OCH3), 3.99 (m, 2H, H-2"), 5.38 (m, 2 H, H-1"), 6.80 (s, 2H, H-6' + H-2'), 7.26 (dd, 1H, H-9, ^{3}J = 6,1 Hz, ^{4}J = 0,7 Hz), 7.91 (s, 1H, H-12), 7,95 (dd, 1H, H-1, ^{3}J = 5,6 Hz, ^{4}J = 0,7 Hz), 8.59 (d, 1H, H-10, ^{3}J = 6,1 Hz), 8.77 (d, 1H, H-2, ^{3}J = 5,6 Hz), 9.69 (s, 1H, H-7), 10.46 (s, 1H, H-4). ^{13}C NMR (100 MHz, DMSO + CDCl3): δ/ppm = 55.5 (2C), 58.3 (2C), 60.1, 61.1, 68.6, 106.0 (2C), 115.3, 116.7, 119.5, 120.4, 124.1, 124.7, 126.2, 136.9, 137.7, 138.3, 140.0, 143.1, 143.6, 144.9, 147.1, 148.3, 149.3, 154.1, 158.7. IR (ATR): ν˜/cm–1 = 3088, 1699, 1647, 1456, 1417, 1386, 1338, 1149, 1035, 1001, 862, 767. MS (ESI): m/z = 501.2 $[M+H]^+$, 440.1, 414.1 $[P8\text{-}DO+H]^+$, 251.0 $[M+2H]^{++}$

HPLC purity: 98.09 %

HPLC

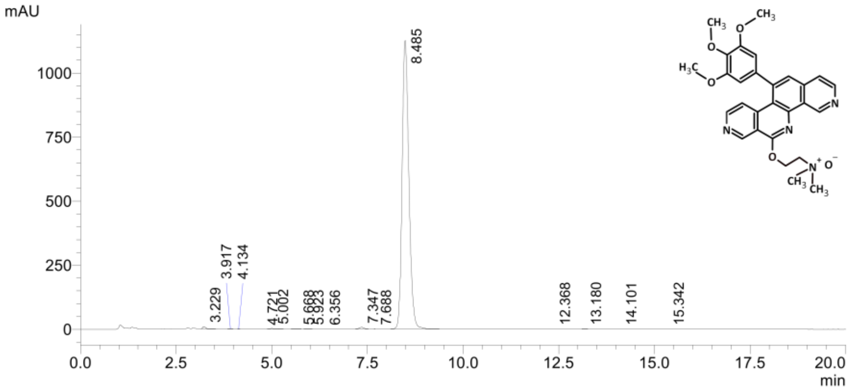

PDA Ch2 260nm

Peak#	Ret. Time	Area	Height
1	3.229	51563	7865
2	3.917	10350	1360
3	4.134	6083	522
4	4.721	3353	366
5	5.002	46620	3080
6	5.668	17057	1157
7	5.923	14793	1111
8	6.356	4269	259
9	7.347	76329	6752
10	7.688	6753	593
11	8.485	13977586	1122180
12	12.368	3200	150
13	13.180	18818	922
14	14.101	10084	480
15	15.342	2950	120
Total		14249809	1146920

LC-MS

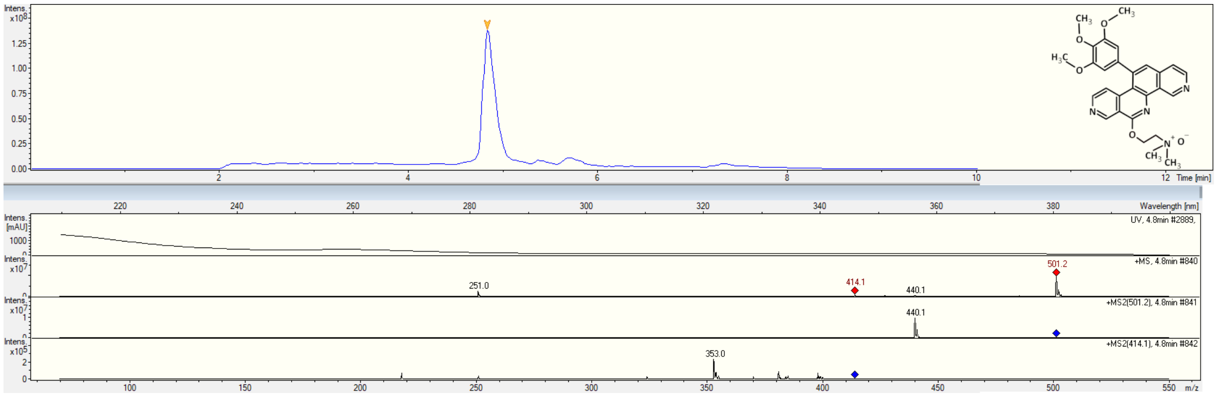

H-NMR

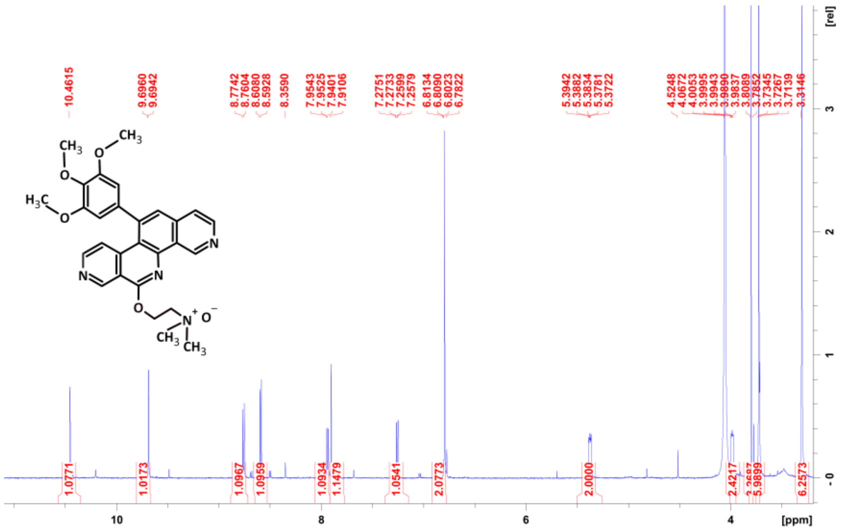

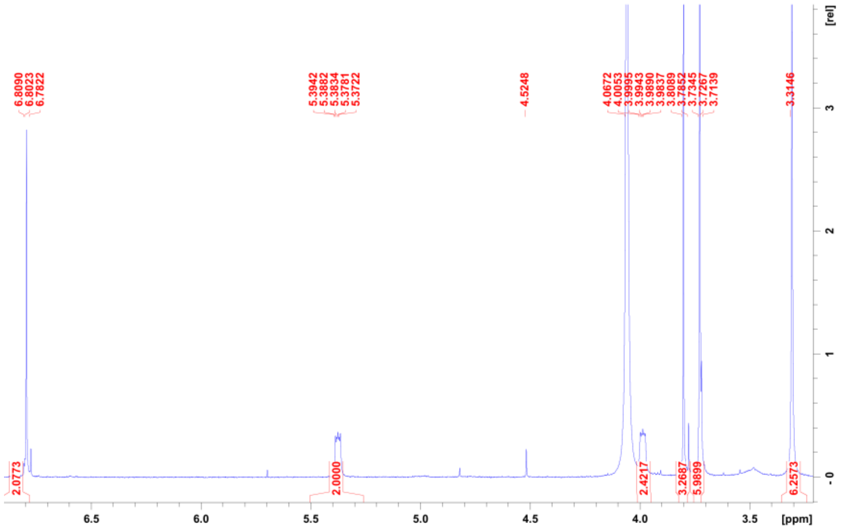

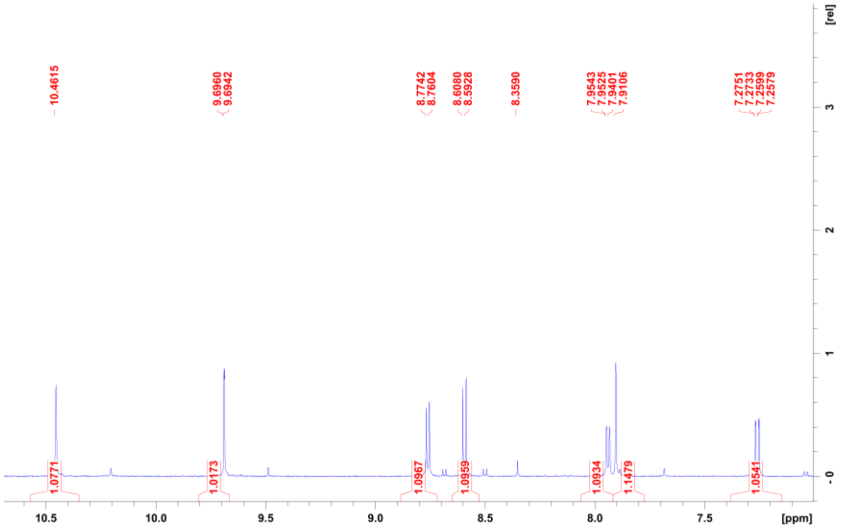

C-NMR

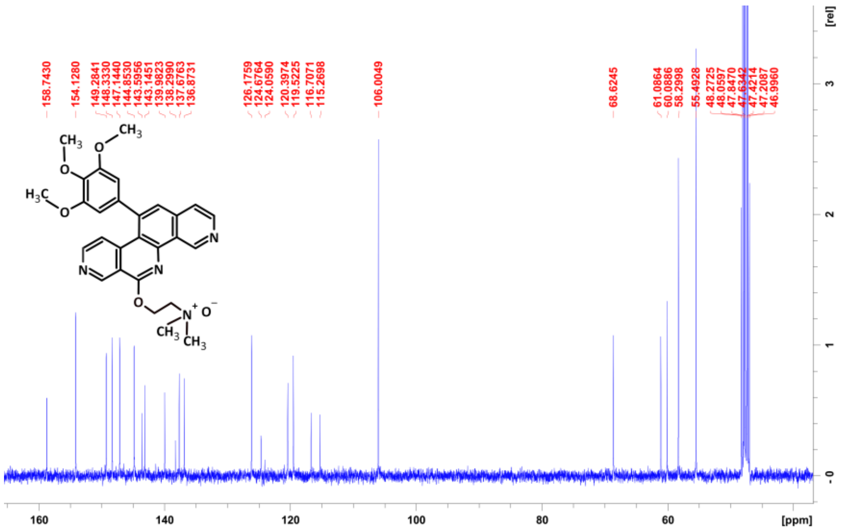

P8-DO (IUPAC: 11-(3,4,5-Trimethoxyphenyl)-5,6-dihydropyrido[3,4-c][1,9]phenanthrolin-6-one. SMILES: COc1cc(cc(c1OC)OC)c2cc3ccncc3c4c2c5ccncc5c-(=O)[nH]4)

All spectroscopic results were in accordance with data from Meier *et al*[2] who published this substance first.

HPLC purity: 98.99 %

HPLC

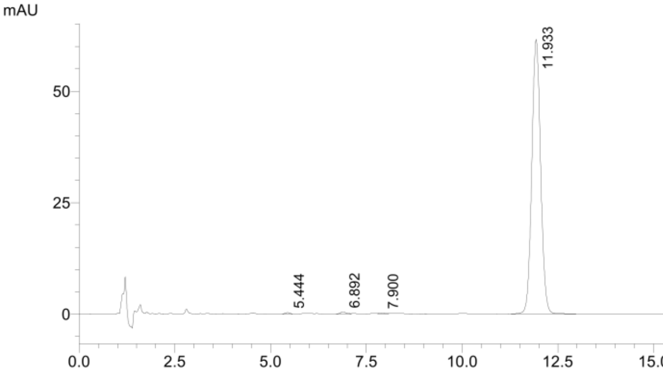

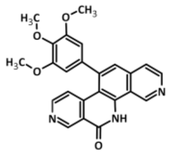

PDA Ch2 260nm

Peak#	Ret. Time	Area	Height
1	5.444	2635	311
2	6.892	4721	430
3	7.900	2779	210
4	11.933	992196	61458
Total		1002330	62409

LC-MS

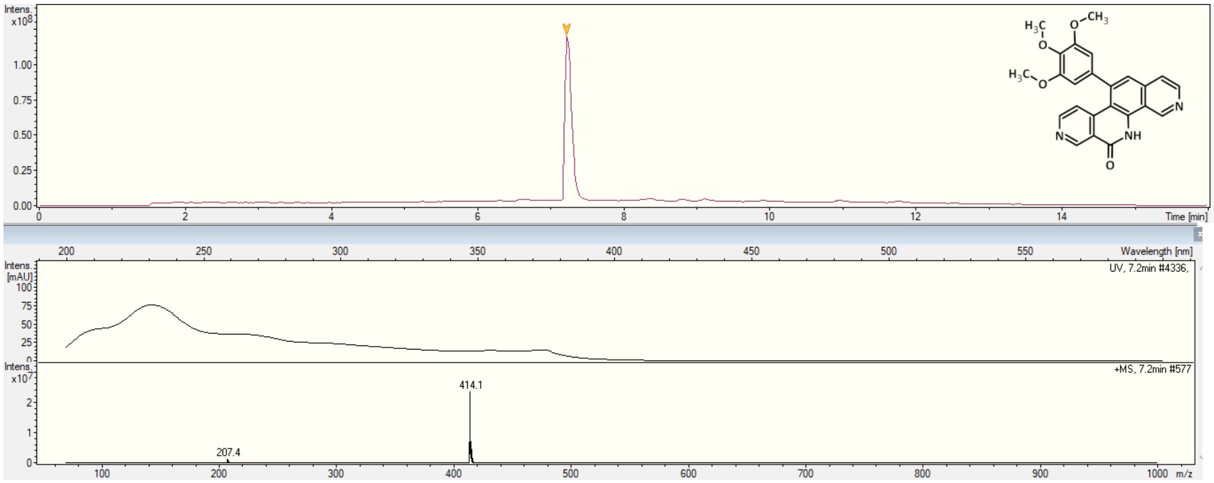

P8-D6 (IUPAC: 6-(N,N-dimethyl-2-aminoethoxy)-11-(3,4,5-trimethoxyphenyl)pyrido[3,4-c][1,9]phenanthroline. SMILES: COc5cc(c3cc1ccncc1c4nc(OCCN(C)C)c2cnccc2c34)cc(OC)c5OC)

All spectroscopic results were in accordance with data from Meier *et al*[2] who published this substance first.

HPLC purity: 97.14 %

HPLC

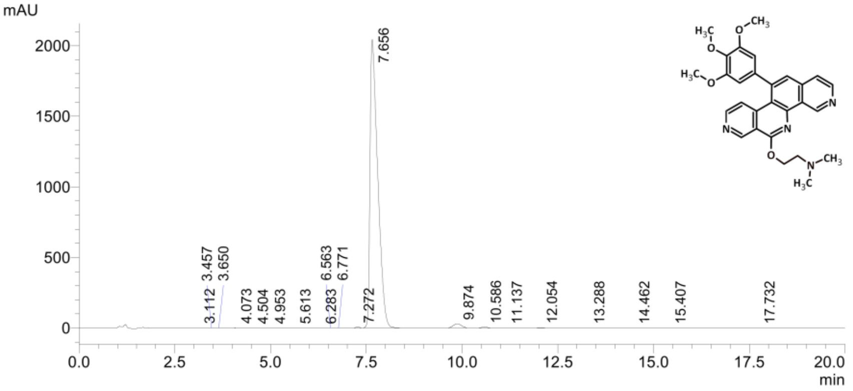

PDA Ch2 260nm

Peak#	Ret. Time	Area	Height
1	3.112	6888	1167
2	3.457	2921	400
3	3.650	12393	1232
4	4.073	25045	2629
5	4.504	4521	492
6	4.953	3287	398
7	5.613	10260	756
8	6.283	4138	379
9	6.563	3712	350
10	6.771	6327	491
11	7.272	62349	5902
12	7.656	28773384	2037937
13	9.874	475027	29578
14	10.586	101962	5408
15	11.137	4492	202
16	12.054	71890	4220
17	13.288	16925	788
18	14.462	5377	281
19	15.407	18630	576
20	17.732	10318	320
Total		29619847	2093505

LC-MS

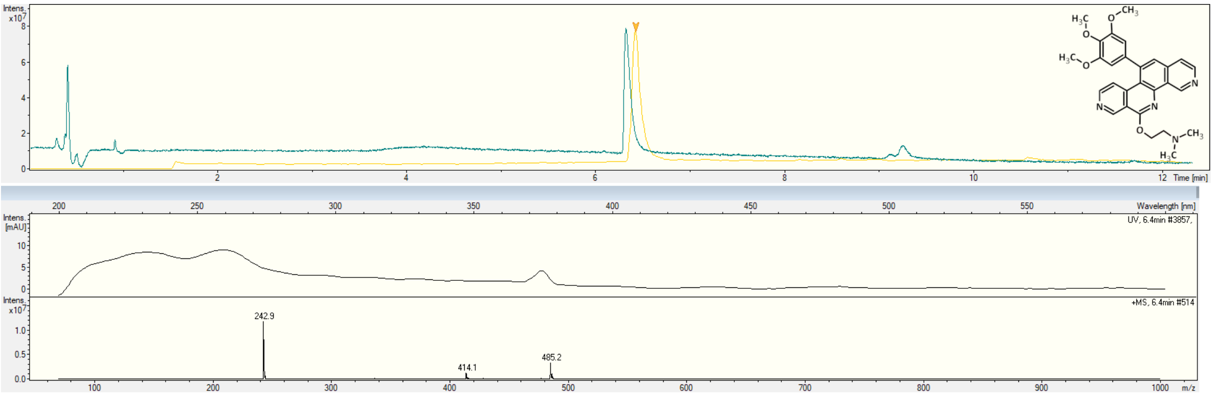

3. Methods

Enzymatic material:

Table S 3: Overview of tissue and species for in vitro incubation.

	species	tissue	subcellular fractions	product	company
our lab	human	liver	PNS (n = 81)		
	rat	liver	PNS		
		lungs	PNS		
		kidney	PNS		
		spleen	PNS		
		colon	PNS		
	pig	liver	PNS		
			S9		
			Microsomes		
	mouse	liver	PNS		
		lungs	PNS		
		kidney	PNS		
commercial	human	liver	S9	H2630.S9	Xeno-tech
			S9 (female)	H1500.S9	
			Microsomes	H2620	
	rat	liver	S9	R1500.S9	
			Microsomes	R1500	
	human	recombinant	FMO 3	Supersomes FMO3	Corning
			CYP 1A2	EasyCyp CYP1A2 High Reductase	Xeno-tech
			CYP 2B6	EasyCyp CYP2B6 Reductase + B5	
			CYP 2C8	EasyCyp CYP2C8 Reductase + B5	
			CYP2C9	EasyCyp CYP2C9*3 Reductase + B5	
			CYP 2C19	EasyCyp CYP2C19 Reductase + B5	
			CYP 2D6	EasyCyp CYP2D6 High Reductase	
			CYP 3A4	EasyCyp CYP3A4 (Low) Reductase + B5	

Postnuclear supernatant (PNS); Xenotech (Xenotech, Tebubio GmbH, Offenbach, Germany); Corning (Corning, New York, USA)

Scheme for the pharmacokinetic analysis

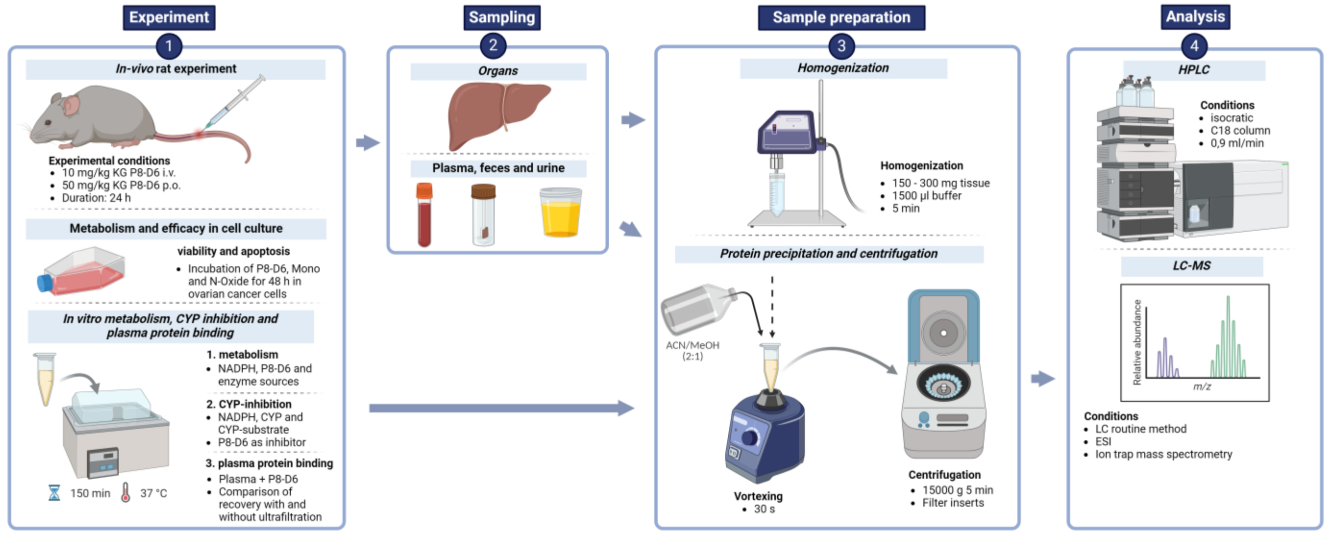

Figure S 5. Preparation scheme

Preparation scheme of the *in vivo* und *in vitro* pharmacokinetic studies. (1) incubation studies, (2) special sampling, (3) sample preparation and purification (4) identification and quantification using analytical methods. This figure was created using Biorender.com

Pharmacokinetic analysis of concentration - time data

Data was performed using GraphPad Prism version 9.0 (GraphPad Software, USA). The time of the maximum concentration (t_{max}) and maximum plasma concentration (c_{max}) was determined from the plasma concentration–time plots. The area under the plasma concentration–time curve (AUC 0–600min, mmol·min/mL) was calculated using the linear trapezoidal method.

To calculate the critical parameters for the one/two compartment models, the method of residuals ("feathering") was performed according to [3–7]. Therefore, this mathematical method can be seen as accepted and validated. The following assumptions were made: the plasma concentration after oral administration would act according to a bateman kinetic while the intravenous bolus application would be described best by an open two compartment model. In both cases, the kinetic is described as a composed equation of a first oder invasion (absorption/distribution) and a first oder elimination rate[3].

In the first step all detected plasma concentrations (c_p) were logarithmized (= ln c_p). Afterwards, a linear regression of all data points from the terminal elimination phase was performed to calculate fictive plasma concentrations for all data points (= cp^{late}) and the model parameters for the elimination phase (R^2 > 0.92 was accepted). Next, all fictive plasma concentrations (cp^{late}) were delogarithmized and the initial plasma concentrations c_p were subtracted before these results were logarithmized again (= ln (cp^{late} - c_p)). Finally, a linear regression of all data points from the initial distribution/absorption phase was performed to calculate the model parameters for the initial phase (R^2 > 0.9 was accepted). In summary, this method yields k_a (absorption) and k_e (elimination) as the slope of their respective linear regressions as well as a fictive initial concentration c_0 at the intersection of both linear regressions for the one compartment model. For the two compartment model, deriving from their respective linear regression this method yields both a fictive initial plasma concentration as the intersection with the y-axis for the distribution (A) and elimination phase (B) as well as their respective hybrid (macro) rate constants α (dibstribution) and β (elimination phase) as the slope of each regression.

The following equations were used to calculate the pharmacokinetic parameters: [3–5,8–11]

The bioavailability of P8-D6 calculated using the following equation:

$$\mathrm{F_{abs,oral}}\ (\%) = \frac{AUC(oral)}{AUC(iv)} * \frac{D(iv)}{D(oral)} * 100$$

The following formulas were used for the oral one-compartment model. The biological half-life was calculated as:

$$t_{\frac{1}{2}} = \frac{\ln(2)}{k_e}$$

The apparent volume of distribution was calculated as:

$$V_{d,abs} = \frac{F * D}{c_0}$$

The clearance was calculated by the following formula:

$$CL_{total} = k_e * V_{d,abs}$$

Additionally, t_{max} and c_{max} were calculated as:

$$t_{max} = \frac{1}{k_a - k_e} * \ln(\frac{k_a}{k_e})$$

$$c_{max} = \frac{F * D}{V_d} * e^{-k_{e*}t_{max}}$$

In the two-compartment model for the i.v. administration of P8-D6 the following equations were used. In contrast to the apparent volume of distribution after p.o./enteral administration a central volume of distribution was calculated as:

$$V_c = \frac{Dose(i.v.)}{c_0(Plasma) * BW}$$

The micro constants k10, k12 and k21 were calculated with the following formulas:

$$k_{12} = \frac{A * B * (\alpha - \beta)^2}{c_0 * (A * \beta + B * \alpha)}$$

$$k_{21} = \frac{A * \beta + B * \alpha}{c_0}$$

$$k_{10} = \frac{\alpha * \beta * c_0}{A * \beta + B * \alpha}$$

The apparent and steady-state volume of distribution were calculated as:

$$V_d = V_{d2} = \frac{V_c * k_{12}}{k_{21}}$$

$$V_{ss} = V_c + V_d = V_c * \left(\frac{1 + k_{12}}{k_{21}}\right)$$

And last but not least, the initial (α) and terminal (β) half-life were calculated by these equations:

$$t_{\frac{1}{2}}(\alpha) = \frac{\ln(2)}{\alpha} \qquad t_{1/2}(\beta) = \frac{\ln(2)}{\beta}$$

4. References

[1] Zhang, Y.; Huo, M.; Zhou, J.; Xie, S. PKSolver: An add-in program for pharmacokinetic and pharmacodynamic data analysis in Microsoft Excel. *Computer methods and programs in biomedicine* **2010**, *99*, 306–314. DOI: 10.1016/j.cmpb.2010.01.007.

[2] Meier, C.; Steinhauer, T. N.; Koczian, F.; Plitzko, B.; Jarolim, K.; Girreser, U.; Braig, S.; Marko, D.; Vollmar, A. M.; Clement, B. A Dual Topoisomerase Inhibitor of Intense Pro-Apoptotic and Antileukemic Nature for Cancer Treatment. *ChemMedChem* **2017**, *12*, 347–352. DOI: 10.1002/cmdc.201700026.

[3] Garrett, E. R. The Bateman function revisited: a critical reevaluation of the quantitative expressions to characterize concentrations in the one compartment body model as a function of time with first-order invasion and first-order elimination. *Journal of pharmacokinetics and biopharmaceutics* **1994**, *22*, 103–128. DOI: 10.1007/BF02353538.

[4] Macheras, P. Method of residuals: estimation of absorption and elimination rate constants having comparable values. *Biopharmaceutics & drug disposition* **1987**, *8*, 47–56. DOI: 10.1002/bdd.2510080106.

[5] Yáñez, J. A.; Remsberg, C. M.; Sayre, C. L.; Forrest, M. L.; Davies, N. M. Flip-flop pharmacokinetics--delivering a reversal of disposition: challenges and opportunities during drug development. *Therapeutic delivery* **2011**, *2*, 643–672. DOI: 10.4155/tde.11.19.

[6] Garrett, E. R. Simplified methods for the evaluation of the parameters of the time course of plasma concentration in the one-compartment body model with first-order invasion and first-order drug elimination including methods for ascertaining when such rate constants are equal. *Journal of pharmacokinetics and biopharmaceutics* **1993**, *21*, 689–734. DOI: 10.1007/BF01113501.

[7] Curry, S. H.; Whelpton, R. *Introduction to Drug Disposition and Pharmacokinetics*; Wiley, 2016.

[8] Korzekwa, K.; Nagar, S. Process and System Clearances in Pharmacokinetic Models: Our Basic Clearance Concepts Are Correct. *Drug metabolism and disposition: the biological fate of chemicals* **2023**, *51*, 532–542. DOI: 10.1124/dmd.122.001060.

[9] Perlin, E.; Taylor, R. E.; Peck, C. Clinical pharmacokinetics: a simplified approach, Part 1. *Journal of the National Medical Association* **1985**, *77*, 475–482.

[10] Wagner, J. G. Linear pharmacokinetic equations allowing direct calculation of many needed pharmacokinetic parameters from the coefficients and exponents of polyexponential equations which have been fitted to the data. *Journal of pharmacokinetics and biopharmaceutics* **1976**, *4*, 443–467. DOI: 10.1007/BF01062831.
[11] Yu, R.-H.; Cao, Y.-X. A method to determine pharmacokinetic parameters based on andante constant-rate intravenous infusion. *Scientific reports* **2017**, *7*, 13279. DOI: 10.1038/s41598-017-13437-6.
